# Environmental microbiota transfer from forest soil into urban homes: a proof-of-principle study

**DOI:** 10.1186/s40168-026-02352-6

**Published:** 2026-03-25

**Authors:** Martin Täubel, Megan S. Hill, Sarah Allard, Jack A. Gilbert, Maria Valkonen, Anne M. Karvonen, Asko Vepsäläinen, Juha Pekkanen, Pirkka V. Kirjavainen

**Affiliations:** 1 Lifestyles and Living Environments Unit, Department Public Health, Finnish Institute for Health and Welfare, Kuopio, Finland; 2 Department of Civil Engineering, Aalto University, Espoo, Finland; 3 Department of Pediatrics, University of California San Diego School of Medicine, La Jolla, San Diego, CA USA; 4 Center for Marine Biotechnology and Biomedicine, Scripps Institution of Oceanography, University of California, La Jolla, San Diego, CA USA; 5 Soil Health Center, Scripps Institution of Oceanography, University of California, La Jolla, San Diego, CA USA; 6 Department of Public Health, University of Helsinki, Helsinki, Finland; 7 Institute of Public Health and Clinical Nutrition, University of Eastern Finland, Kuopio, Finland

**Keywords:** Indoor microbiome, Microbiota transfer, Asthma prevention, Environmental microbiota interventions

## Abstract

**Background:**

Urban lifestyles are characterized by reduced encounters of environmental microbe stimuli that activate immunoregulatory pathways. This has been linked to an increased risk of inflammatory diseases, asthma, and allergies. A potential preventative solution is to modify indoor microbial exposures toward health-promoting interactions. Here, we test the feasibility of environmental microbiota transfers into urban homes and quantify the spatiotemporal impact on the built environment microbiota.

**Methods:**

House dust microbiota of six Finnish homes was monitored over a 20-week period by collecting settled dust from infant (IBZs) and adult breathing zones (ABZs) and floor dust from different home locations. Microbiota in dust samples was characterized using qPCR and amplicon sequencing of the bacterial and archaeal 16S rRNA gene and fungal ITS1 region. Microbiota transfers were performed with repeated seeding of forest soil onto rugs placed in the home entryway.

**Results:**

We observed significant, post-intervention increases in the relative abundances of forest soil bacteria in house dust. The magnitude of effect was influenced by building characteristics, spatiotemporal dynamics, and occupant dynamics and was greatest in a home with comparably little additional microbial influx—a home with no pets, low occupancy, and mechanical ventilation. The most pronounced effect was observed in settled dust close to the soil-seeded rugs at IBZ, within the first 2 weeks after each seeding event, though the soil-associated bacterial signal also extended spatially into the living areas of the homes. Increases in bacterial diversity and an asthma protective microbiota index, as well as decreases in the proportion of human-sourced bacteria, were also observed, but only in airborne dust close to the soil-seeded rug. Effects on *fungal* microbiota or on the bacterial and fungal *loads* in house dust were inconsistent.

**Conclusions:**

We demonstrate that a simple soil-to-rug intervention can modify the bacterial microbiota in airborne particulate matter in residential homes. The introduction of specific environmental soil microbes was most pronounced closest to the source, which is relevant when targeting infant inhalation exposure. While this approach is promising, specifically in highly urbanized settings, dosage and composition of environmental microbiota additions to reach health benefits require further study.

Video Abstract

**Supplementary Information:**

The online version contains supplementary material available at 10.1186/s40168-026-02352-6.

## Background

The construction and location of built environments strongly shape our interactions with microorganisms, particularly as they influence our general proximity to various types of green spaces and natural environments. For example, a high percentage of built area around Finnish homes has been associated with lower richness and diversity of the bacterial microbiota in entryway door mats when comparing rural and urban households [1]. Similarly, urban homes had significantly lower bacterial and fungal concentrations in indoor air, airborne settled dust, and floor dust compared to rural homes in a quantitative, longitudinal assessment of indoor microbes [2]. Further, a study in Belgian residences showed how nearby green space is a determinant of indoor environment microbiota and that the type of greenery around the home differentially impacted bacterial and fungal compositions indoors [3].

The importance of environmental exposures to diverse microorganisms is suggested by the biodiversity hypothesis [4] and other similar [5, 6] nature-focused derivations of the original hygiene hypothesis [7]. These concepts are based on the importance of rich and sufficiently abundant early-life microbial stimulation of immunoregulatory pathways, either directly or via interaction with the development of the human microbiome [8]. A canine study by Lehtimäki et al. [9] demonstrates how the influence of environmental microbes on host microbiota depends on the living environment and lifestyle, and such findings have been further supported in human studies. For example, closeness to forested or agricultural lands can increase the relative abundance of environmental bacteria on the skin [10, 11], greenness in residential areas is positively correlated with upper airway, skin, and gut microbial diversity (e.g., [12, 13]), and growing up on farms promotes healthy infant gut microbiota maturation [14]. As evidenced by a growing body of literature, the removal from these diverse microbial exposures has negative health consequences. This is particularly true during early life, where urbanization-associated changes in infant microbiota may elevate the risk of asthma and phenotypic expression of atopy [15].

Dysregulation of the immune system is a major health concern, contributing to the rise of chronic inflammatory diseases such as asthma, autoimmune disorders, and allergic conditions, which significantly affect overall well-being and quality of life [16]. Asthma alone caused nearly half a million deaths worldwide in 2019 [17], and the rates of these diseases have increased more rapidly in urban environments [18]. Qualitative and quantitative specifics of the indoor microbiota composition have been associated with suppressed proinflammatory cytokine responses against bacterial immunogens that are ubiquitous in household dust and with an increased risk of childhood asthma [19, 20]. Additionally, a deficit of environmental bacteria on the skin has been associated with decreased expression of an anti-inflammatory cytokine IL-10 and has been shown to correlate with an increased risk of developing atopy and asthma in children [10, 11, 21]. The connection between environment, indoor dust microbiota, and anti-inflammatory mediated protection against asthma has been demonstrated convincingly in a US study comparing Amish and Hutterite communities [22]. These communities have similar lifestyles and genetic ancestries, but the Amish follow traditional family farms’ practices, while the Hutterites have large communal farms assisted with modern technology. This culminates in deprived indoor dust microbial exposures, lower abundance of immunosuppressive monocytes, more robust cytokine responses to innate bacterial stimulants (lipopolysaccharides, LPS), and higher prevalence of asthma in the Hutterite as compared to the Amish children. The causal link with indoor dust microbiota was supported in an animal model of allergic asthma where the dust from the Amish but not Hutterite homes inhibited ovalbumin-induced broncho-alveolar eosinophilia in mice [23].

Our earlier research findings show that the home dust microbiota represents a modifiable target in asthma prevention efforts [19]. This has sparked interest in the development of tractable strategies that increase biodiversity indoors, are cost-effective, easy to implement, and inclusive across different populations. Here, we tested the feasibility of environmental microbiota transfer into urban Finnish homes by performing a simple forest soil-on-rug intervention. Soil microbiota was selected as a practical example of a transferable environmental microbial community that could have health relevance, as soil carriage has been suggested to contribute to asthma protective indoor microbiota composition and to promote immunoregulation [10, 24]. We studied the effects of the soil-on-rug intervention on indoor microbiota characteristics and associated spatiotemporal aspects. We hypothesized that the intervention would result in the detection of forest soil-associated microbes in airborne particulate matter (PM), with both temporal and spatial decays of such soil-microbe signals. We, furthermore, anticipated that the intervention would diversify bacterial and fungal taxa in airborne PM, but not significantly increase the microbial loads, due to low amounts of soil introduced to the homes relative to dust and debris from other sources generally accumulating in households.

## Methods

### Study design

**Fig. 1:**
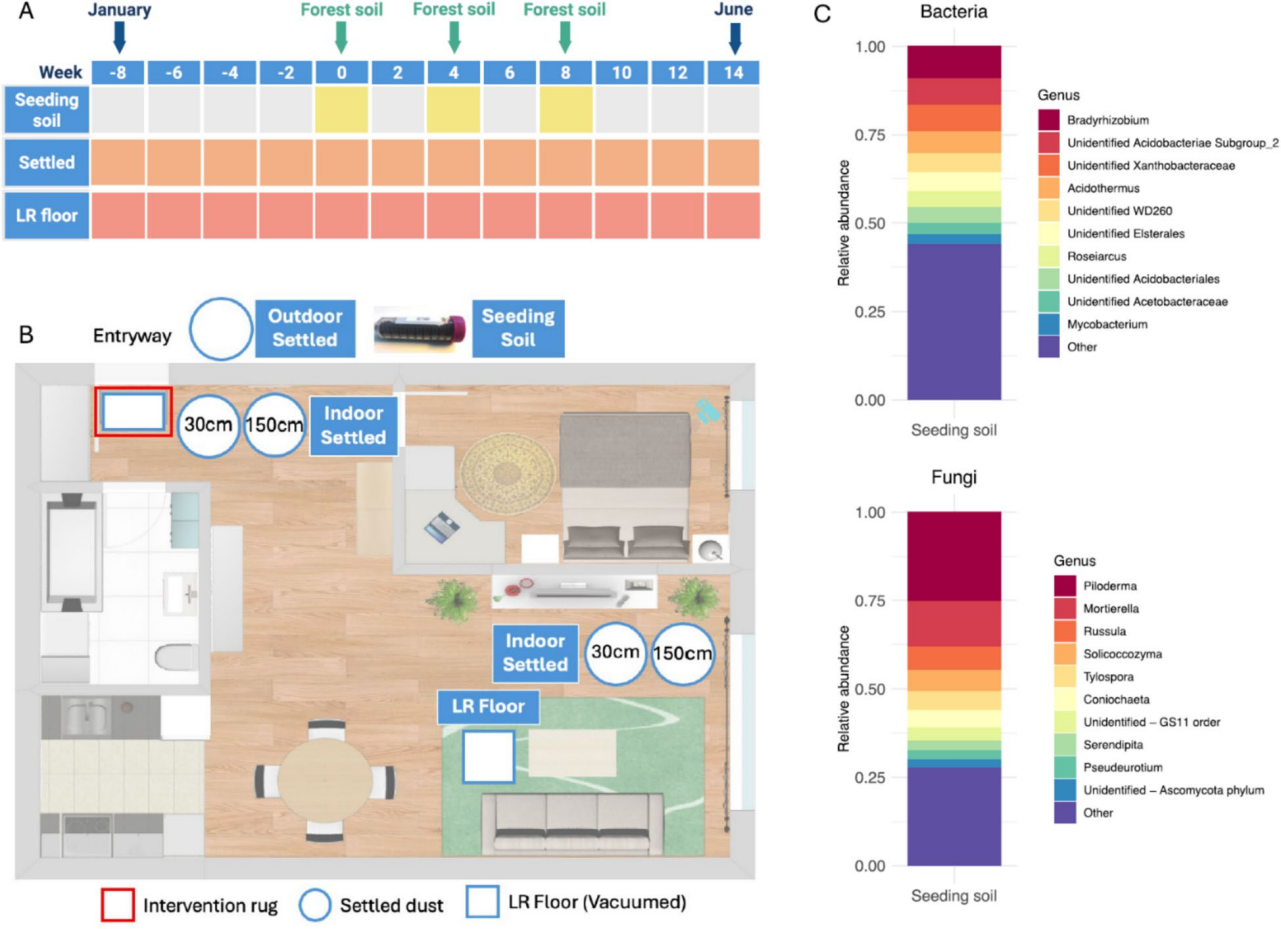
Sampling scheme, study timeline, and forest soil composition. **A** Timeline of sample collection by sample type. Weeks −8 to −2 indicate the baseline sampling period. Forest soil-on-rug seeding interventions were carried out in weeks 0, 4, and 8. Settled dust samples were collected passively over two weeks and include indoor and outdoor locations; living room (LR) floor samples were collected by vacuuming. **B** Sample types are indicated by blue boxes. Indoor settled dust samples (passive deposition of airborne particulate matter into petri dishes) were collected in the entrance area near the intervention rug, as well as in the living area of each home. Sampling heights of 30 cm and 150 cm were selected to represent the infant (IBZs) and adult breathing zones (ABZs). **C** Top ten most abundant bacterial and fungal genus-level taxa identified from the forest soil that was used to seed each intervention rug

To assess changes to the indoor microbiome associated with adding forest soil into homes, the bacterial and fungal microbiota were characterized qualitatively and quantitatively from six residential homes located in the wider Kuopio area in Eastern Finland (*n* = 5 intervention homes, *n* = 1 control home). Samples were collected every two weeks, over a period of 22 weeks from January to June of 2018 (Fig. 1A), and included settled dust collected from four indoor and, where possible, one outdoor location, as well as floor dust from the living room (LR) (Fig. 1B). Settled dust samples indoors were collected from the entrance area, next to where the intervention rug was placed, and from the living room area in each home, at heights representing infant (IBZs) and adult breathing zones (ABZs), respectively. Baseline samples were collected for 8 weeks. After this 8-week period, rugs that had been placed in the interior of the entryway door were seeded with forest soil at 4-week intervals at 0, 4, and 8 weeks; the control home received the same rug with no soil. Following the final intervention (week 8), the indoor microbiota was monitored for an additional six weeks (Fig. 1A).

Homes included an apartment and single-family homes that varied in age (built between 1950 and 2000), size (45 m^2^–120 m^2^), and ventilation type, including natural ventilation, mechanically supported exhaust, or fully mechanical ventilation with air filtration. Occupancy varied from two persons to families of five and from no pets to three dogs, and each home was placed into one of two categories that reflected the level of urbanization: semi-urban or semi-rural. Though both regions are characterized by proximity to greenery, semi-urban homes are located in the outer region of Kuopio with full infrastructure, whereas semi-rural homes are further from the urban center (> 10 km), have less infrastructure, and population density is comparably lower. A summary of all building, occupancy, and location characteristics of the individual homes is presented in Table 1.

**Table 1:** Characteristics of the study homes, occupant demographics, and sample sites. “Living room distance” in the last column indicates the proximity of living room settled dust samplers to where the soil rug was positioned in the entrance area

Home	Home type	Year of construction	Region	House size	Ventilation	Occupants	Pets	Living room distance; flooring type
1	Apartment, 8th floor	2000	Semi-urban	45 m^2^	Full mechanical	1 adult; 1 child every 2 weeks	Occasionally 1 dog	3 m; smooth floor
2	Detached house, one floor	1989	Semi-rural	120 m^2^	Full mechanical	2 adults, 2 children	3 dogs	6 m; smooth floor and rugs
3	Detached house, one floor	1998	Semi-rural	100 m^2^	Mechanically supported exhaust	2 adults, 1 child	3 dogs	7 m; smooth floor and rugs
4	Detached house, three floors	1952	Semi-urban	180 m^2^	Natural	2 adults, 3 children	None	3 m; smooth floor and rugs
5	Detached house, one floor	1985	Semi-urban	105 m^2^	Mechanically supported exhaust	2 adults, 3 children	None	8 m; smooth floor and rugs
Control	Semi-detached house, one floor	1980	Semi-urban	114 m^2^	Mechanically supported exhaust	2 adults, 2 children	1 dog	3 m; smooth floor

### Forest soil collection and rug seeding

Soil was collected in February 2018, during full snow cover from the Neulaniemi recreational area in Kuopio, Eastern Finland. The sampling area is in the boreal coniferous vegetation zone, and the specific sampling site is in a forest dominated by spruce and birch (Latitude: 62.9033, Longitude: 27.5975). Kuopio is in the subarctic climate zone, according to Köppen climate classification, and meteorological conditions at the time of sampling (27.2.2018, 12.15) were sunny, −16.8 °C, RH 60%. At the sampling site, 70 cm of the snow cover was removed and approximately 10 cm of the topsoil from a 50 × 50 cm area was harvested into resealable plastic bags. The sampled soil was stored overnight at 4 °C and homogenized through a sterile sieve (pore size 1 mm × 2 mm) thereafter. Aliquots of 15 g were weighed into 50 mL sterile screw cap vials. Soil aliquots (henceforth referred to as “seeding soil”) were stored at −20 °C between each rug seeding event. In addition, five subsamples of 50 mg were weighed into DNA extraction tubes for baseline assessment of bacterial and fungal communities. Additionally, seeding soil was sequenced at each intervention timepoint to ensure retention of a stable community signature and to allow for direct comparisons between each soil aliquot and the corresponding home microbiota (Fig. 1). We acknowledge that freezing of the seeding soil will have likely reduced the viability of microbes in the samples. However, it was not of the essence in this study to transfer unaltered forest soil into the study homes or to maintain the viability of the soil microbes. In turn, we considered it important to maintain a stable microbial soil signal in the repeated seeding events over the entire duration of this multi-month study, which is why freezing of the seeding soil was necessary.

The intervention rugs used in this study are commercially available, 80 cm × 200 cm flat-woven rugs, with a thickness of 5 mm and a surface density of 1385 g/m^2^. The surface material is 100% polypropylene, with a synthetic rubber backing. The rugs were new and thoroughly vacuumed before the first use but not sanitized or otherwise treated. Soil inoculation and rug sampling were conducted in a dedicated laboratory facility at the Finnish Institute for Health and Welfare (THL), Kuopio, Finland. For each seeding event, a 15 g aliquot was thawed and evenly applied to the entire surface of the carpet through a sterile sieve (pore size 1 mm × 2 mm). The top was then covered with kraft paper to avoid contamination, and the soil was embedded into the rug with a plate compactor (75 kg Swepac, 230 V), operating at a vibration frequency of 88 Hz for 2 min, while being moved over the entire surface. This soil embedding approach was inspired by work done by Shorter [25], and previous research has suggested that embedding dust using a plate impactor provides a dust distribution in rugs that mimics rugs from homes [26]. Within 48 h of embedding, rugs were covered with kraft paper, rolled up, placed into a plastic bag, and transported to each study home. Prior to reseeding, this process was reversed (i.e., in the home, the top surface of the rugs was covered with kraft paper, the rug was rolled up by the study participants, placed into a plastic bag, and transferred to the laboratory) for sampling and reinoculation at THL facilities.

### Sampling

At all timepoints, airborne dust that settled onto sterile petri dishes and living room (LR) floor dust samples were collected (Fig. 1B). Airborne, settled dust was collected using the petri-dish approach, as described in Adams et al. 2015 [27]. Passive collection of particulate matter on a standardized surface was selected as the primary sampling approach, as it represents airborne microbial exposure integrated over a specified sample collection period, overcoming the known limitations of short-term, active air sampling, with respect to temporal variability [2]. Sterile petri dishes (four replicates per sampling location) were placed in two areas within each home, in the entryway in immediate proximity to the intervention rug and in the living room of the home, at heights of 30 cm and 150 cm. These two heights were chosen as proxies for typical infant (IBZs) and adult breathing zones (ABZs), respectively. Where feasible, settled dust was also collected outdoors with the same approach from a protected location, typically the terrace. All petri dishes were exposed for two weeks and then closed and transported to the laboratory by the study participants or a field worker, following detailed instructions. The dishes were individually sealed with parafilm and stored in a dry and dark location at room temperature until sample processing within one week. Sampling of floor dust represents the most common sampling approach in epidemiological studies that have interest in the effects of indoor microbial exposures on residents’ respiratory and allergic health and, therefore, was performed here to complement the settled dust collection. Floor dust samples were also collected biweekly by either the study participants or a field worker from the living room of each study home. When carpet was present, 1 m^2^ of the LR rug was vacuumed; otherwise, 4 m^2^ of smooth flooring was vacuumed. LR floor dust was collected by vacuuming for 2 min with the family’s personal vacuum cleaner equipped with a clean nylon dust sampling sock, as described previously [2]. Floor dust samples were collected after completion of the settled dust sampling period and before opening the new petri dishes, so as not to affect settled dust sampling.

Finally, the study participants were asked to fill in a sampling questionnaire at each 2-week interval, including information on occupancy, sampling durations, irregularities during sampling, vacuum cleaning in the home, an estimate of how frequently the rug was walked over daily, and whether the rug was perceived as a disturbance by the occupants. All these aspects remained relatively consistent within the homes throughout the monitoring period (data not shown). Importantly, the study participants reported that the soil-seeded carpet was walked over 20 or more times daily in all cases and periods, except for home 1, where the frequency ranged between 10 and 20 times per day.

### Sample processing and DNA extraction

Settled dust was collected from the sterile petri dishes with a sterile cotton swab that was wetted in a buffer solution (sterilized water and 0.05% Tween 20). The insides of the lids and the entire bottom of all four petri dishes from each sample location were sampled with one swab, which then was clipped with sterile scissors directly into a 2 mL screw cap vial containing 0.5 g of sterile 212–300 nm glass beads (Sigma-Aldrich, St. Louis, MO, USA). LR floor dust and the intervention rug samples were sieved through a sterile strainer (pore size 1 × 2 mm) for homogenization and to exclude larger objects that might interfere with sample extraction. A target amount of 20–25 mg of the dust was weighed into 2 mL screw cap vials that contained 200 mg of sterile glass beads. Similarly, when each intervention rug was seeded at the THL laboratory, three separate 50 mg samples of the seeding soil were weighed into sterile 2 mL screw cap vials containing glass beads. All dust and soil samples were stored at −20 °C until DNA extraction.

DNA extraction and clean-up were performed with a Chemagic DNA Plant kit (PerkinElmer chemagen Technology GmbH, Germany), following an initial bead beating step. Briefly, 400 µL lysis buffer and 10 µL salmon testis DNA internal standard suspension (Sigma-Aldrich Co., USA) [28] were added to each tube. Mechanical cell disruption was then performed using a MiniBeadbeater-16 (Biospec Products, Inc., USA) at maximum speed for 1 min. The DNA was then purified from the suspension on a KingFisher™ mL DNA extraction robot (Thermo Fisher Scientific, Inc., Finland), following the Chemagic DNA Plant Kit protocol. Blank petri dish samples, negative controls, reagent controls from DNA extraction, and in-house prepared bacterial and fungal mock communities [29, 30] were included periodically throughout the DNA extraction process. DNA was stored at − 20 °C until sequencing.

### DNA sequencing and bioinformatic analysis

Indoor microbiome communities were characterized by sequencing the bacterial 16S rRNA gene and fungal ITS region from settled, LR floor and rug dust, as well as seeding soil aliquots. The DNA extracts were shipped frozen to the sequencing service partner LGC Genomics (Germany). The V4–V5 region of the bacterial and archaeal 16S rRNA gene was amplified using 515F/806R primers [31], and the fungal ITS1 region was amplified using standard ITS1F/ITS2 primers [32]. The PCR conditions and protocol were similar to the description detailed in Dockx et al. 2021 [3], with the exception that 30 PCR cycles for 16S amplification and 35 cycles for ITS amplification were run. Sequencing was performed on an Illumina MiSeq with V3 chemistry, producing paired end reads with a length of 300 bp. The libraries were demultiplexed, and all sequence reads were processed with custom Python v2.7.6 scripts that sorted reads by sample and removed the barcodes and amplicon primer sequences. The 16S and ITS sequences were trimmed with the Fastx-toolkit (version 0.0.13; https://github.com/agordon/fastx_toolkit), and cutadapt (version 2.1; [33]) was used to remove the short length reads below 100 bp. The FIGARO python-based application maxEE [34] was used to assess the optimal parameters for quality and truncation criteria: truncation of bacteria (165,186) and fungi (222,151). Reads were then dereplicated and amplicon sequence variants (ASVs) were assigned with the dada2 core sample inference algorithm [35]. Forward and reverse reads were merged, and sequences that were longer or shorter than the expected length due to non-specific primers were removed. Finally, chimeras were removed, and taxonomy was assigned with the SILVA database (version 138; [36]) for bacteria and the UNITE database for fungi (version 8.2; [37]). Downstream post-processing included the removal of ASVs identified as non-bacterial/archaeal (for 16S), non-fungal (for ITS), mitochondria, eukaryote, and chloroplast, as well as singletons. Contaminant ASVs, as identified from the sequencing controls, were removed from the bacterial data with the isContaminant function of the Decontam package (version 1.2) [38], using the “prevalence” method and a probability threshold of 0.5. This method compares prevalence of detection of an ASV in control versus experimental samples and flags ASVs that occur at higher prevalence in the controls, as to be expected from reagent and other contaminants. In this decontamination step, 12 swab blanks were included as controls to compare ASV prevalence. Using this approach, a total of 64 bacterial and 12 fungal ASVs were removed prior to downstream analyses. Bacterial and fungal samples were rarefied to 2000 reads per sample, resulting in *n* = 395 dust and *n* = 37 seeding soil samples. All statistical analyses and visualizations were conducted/produced in the R environment, using the phyloseq, vegan, dplyr, ggplot2, ggpubr, ggthemes, and patchwork packages [39–47], and settled dust and LR floor dust were considered separately.

Chao1 index for estimated species richness and Shannon diversity were calculated from the rarefied datasets, and differences in alpha diversity were compared with either Wilcoxon signed-rank or Kruskal–Wallis tests [48, 49]. To understand the compositional differences between communities, zero values were addressed by adding a pseudocount of 1 prior to centered log-ratio (CLR) transformation [50, 51], and dissimilarity matrices were calculated based on Aitchison distance [50, 52]. Ordinations were visualized with Principal Coordinates Analysis (PCoA) plots, and statistical differences were compared with permutational multivariate analysis of variance (PERMANOVA) tests. Additionally, the relative influence of home characteristics and occupancy patterns on microbial community composition was assessed using multivariate PERMANOVA tests for both bacteria and fungi. Marginal testing was used to evaluate each variable independently, accounting for the effects of other variables within the model. Variables of interest included home type (apartment or single family), home size (m^2^), type of ventilation, number of occupants, whether there was a dog in the home, region (i.e., semi-urban vs. semi-rural), and intervention period. For pre- and post-intervention comparisons, only homes that received the intervention were considered.

Further, to understand if the home microbiota become more similar to the soil communities after each intervention (i.e., can a soil signature be detected), beta distances were calculated with the Bray–Curtis dissimilarity metric between the seeding soil and settled dust samples collected over time and in different locations, as defined in Kodera et al. 2023 [53]. For this analysis, seeding soils from each individual home were averaged and used as the matching baseline community value. For example, distances calculated from seeding soils characterized at *T* = 0, 4, and 8 weeks for Home 1 were averaged and then compared to all Home 1 settled dust samples. Beta distances range between 0 and 1, where a value of 0 indicates community similarity and 1 indicates community dissimilarity. Bacterial and fungal samples were compared using the same methods but considered separately.

Additionally, the relative contribution of microbes at each sample site was calculated using three source-tracking indices: a soil source index (SSI), human source proxy (HSP) [54], and the farm-home resembling microbiota index (FaRMI) [19]). The SSI is a metric to determine the spread of bacteria and fungi sourced from seeding soil samples, similar to the beta distances analysis described above. This metric was calculated as the sum of the relative abundances of the 19 most abundant bacterial taxa and 28 most abundant fungal taxa that were detected in all seeding soil aliquots (Supplemental Table 1). The taxa included in the SSI represented 63%–77% of bacterial and 87%–93% of fungal sequences in the seeding soil aliquots. The HSP provides a measurement proxy for the relative bacterial contribution of human occupants in each home and was calculated as the sum of the relative abundances of *Staphylococcus* spp., *Streptococcus* spp., Propionibacteriaceae, Corynebacteriaceae, and Enterobacteriaceae, as identified to be the most common body-associated taxa in Hospodsky et al. [54]. The FaRMI has been shown to be indicative of the asthma-protective potential of the house dust bacterial community [19]. FaRMI was originally calculated as the individual probability score of a sample being from a farm or rural non-farm home, based on logistic regression analysis of four main PCoA axes from generalized UniFrac analysis. FaRMI was applied to the samples of this study using the GLM regression equation including coefficients for percentile ranked relative abundance of selected predictor taxa (*n* = 45 FaRMI defining taxa; Supplemental Table 2) as described previously [19]. SSI, HSP, and FaRMI were calculated for all settled dust and floor dust samples. Spearman’s Rank Correlation Coefficient was used to calculate statistical differences in all indices between sample locations. A paired *t*-test was used to compare differences in these metrics between seeding events and 2 weeks post-intervention (pre-post2w) and between seeding events and 4 weeks post-intervention (pre-post4w) at individual sample locations.

### Quantitative PCR (qPCR) analysis

To quantitatively profile the bacterial and fungal levels in the house dust samples, qPCR analysis was performed using previously published assays for Gram-positive bacteria, Gram-negative bacteria [29], and total fungal DNA [55]. qPCR reactions were performed largely as described in the original publications with minor modifications [2]. A relative quantification method, including standard curves and a salmon testis DNA internal standard, was applied to calculate the numbers of microbial cell equivalents (CE) in each sample, corrected for inhibition and DNA losses during the extraction process [28, 56]. Positive (i.e., bacterial and fungal mock communities) and negative reagent controls, as well as no template controls, were included to confirm the validity of the qPCR reactions.

## Results

### Home microbiota characteristics pre-intervention

**Fig. 2:**
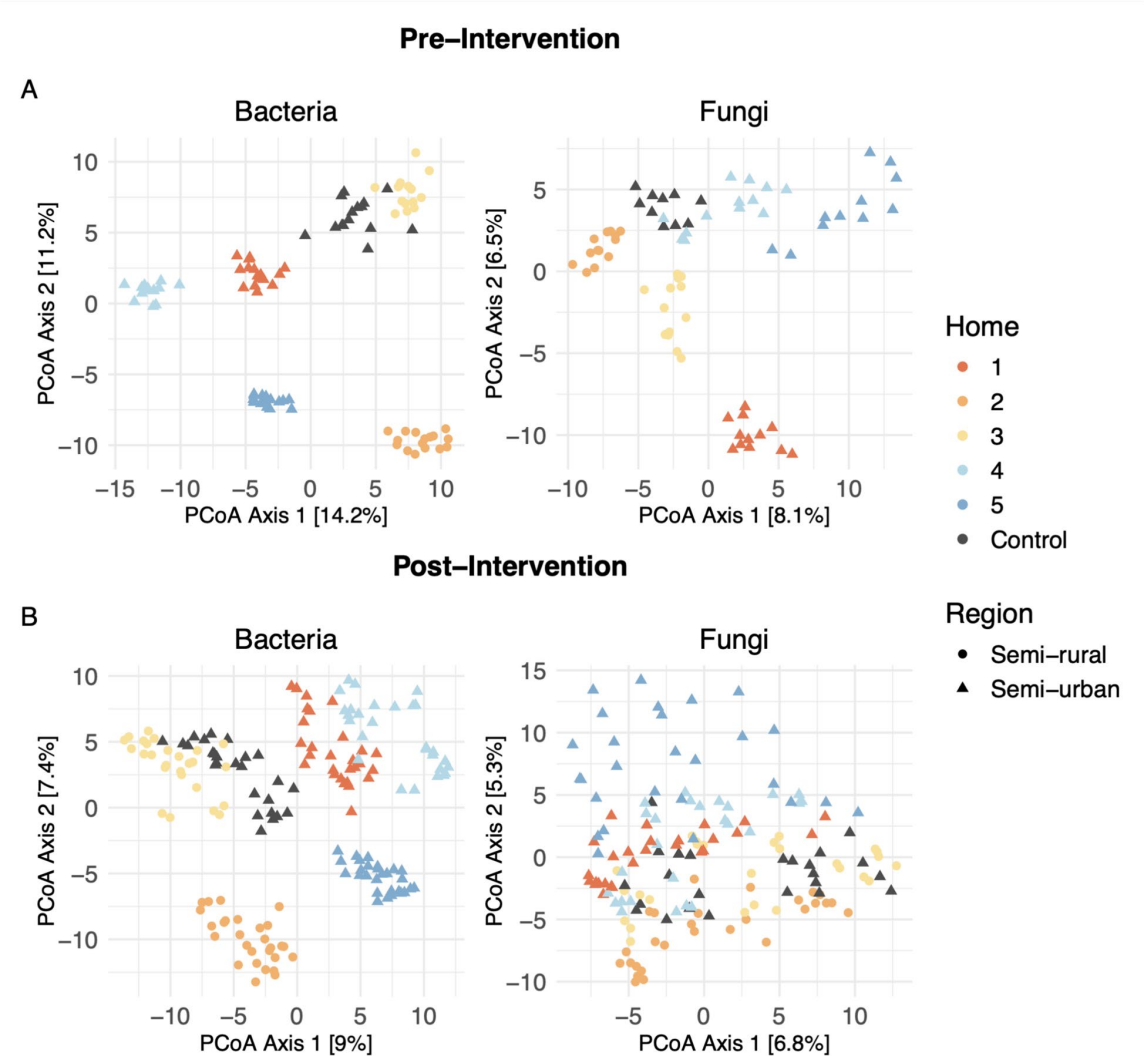
Community composition for pre- and post-intervention time periods for indoor settled dust samples, by home and based on region of location. Homes were distinct from one another both during pre- and post-intervention, with differentiation between region (*p* < 0.001 for all comparisons; Aitchison distance; PERMANOVA). Regional divide is confounded, as both semi-rural homes had dogs, where no semi-urban homes had dogs (except for rare occupation of a dog in home 1)

Settled dust and living room floor dust in the study homes had similar taxonomic profiles. Of the top ten most abundant bacterial genera across the pre-intervention samples, seven were shared between the settled and LR floor dust: *Micrococcus*, *Corynebacterium*, *Staphylococcus*, *Massilia*, *Streptococcus*, *Paracoccus*, and *Kocuria*. Similarly, eight of the top ten most abundant fungal genera across all pre-intervention samples were shared: an unclassified Ascomycota, *Cladosporium*, *Phenoliferia*, *Mrakia*, *Vishniacozyma*, *Penicillium*, *Mycosphaerella*, and *Aspergillus*. However, homes were significantly different from one another in bacterial and fungal community structure (Aitchison Distance; PERMANOVA; settled dust: *p* < 0.001, Fig. 2; LR floor dust: *p* < 0.001; Supplemental Fig. 1). Within homes, there were also significant differences in the bacterial and fungal community structure between the settled and LR floor dust (Aitchison distance; PERMANOVA; bacteria: *p* < 0.001; fungi: *p* < 0.001; Supplemental Fig. 2).

For bacterial communities characterized by 16S amplicons, homes 1, 4, and 5 (semi-urban with no resident dogs, excepting a dog visiting home 1 occasionally) separated along principal coordinate 1 from homes 2, 3, and control (2 and 3 are semi-rural, and all three homes have dogs; Fig. 2; Aitchison distance). For fungal communities, homes 1 and 5 were most unique from the others; however, no home demographics immediately explain the tighter grouping of homes 2, 3, 4, and control (Fig. 2). We further investigated the relative importance of individual home characteristics on microbial communities separately for pre- and post-intervention samples and by sample type (Supplemental Table 3) and found that those explained generally more of the bacterial compared to the fungal and of floor dust compared to settled dust community structure. Due to the low number of homes and high collinearity between variables, it was impossible, however, to reliably determine the strength of individual explanatory factors and to parse their effects (e.g., both semi-rural homes also have three dogs). Thus, the presented data are intended only to serve as a basic description of the house dust microbiota and factors that may contribute to their variability.

**Fig. 3:**
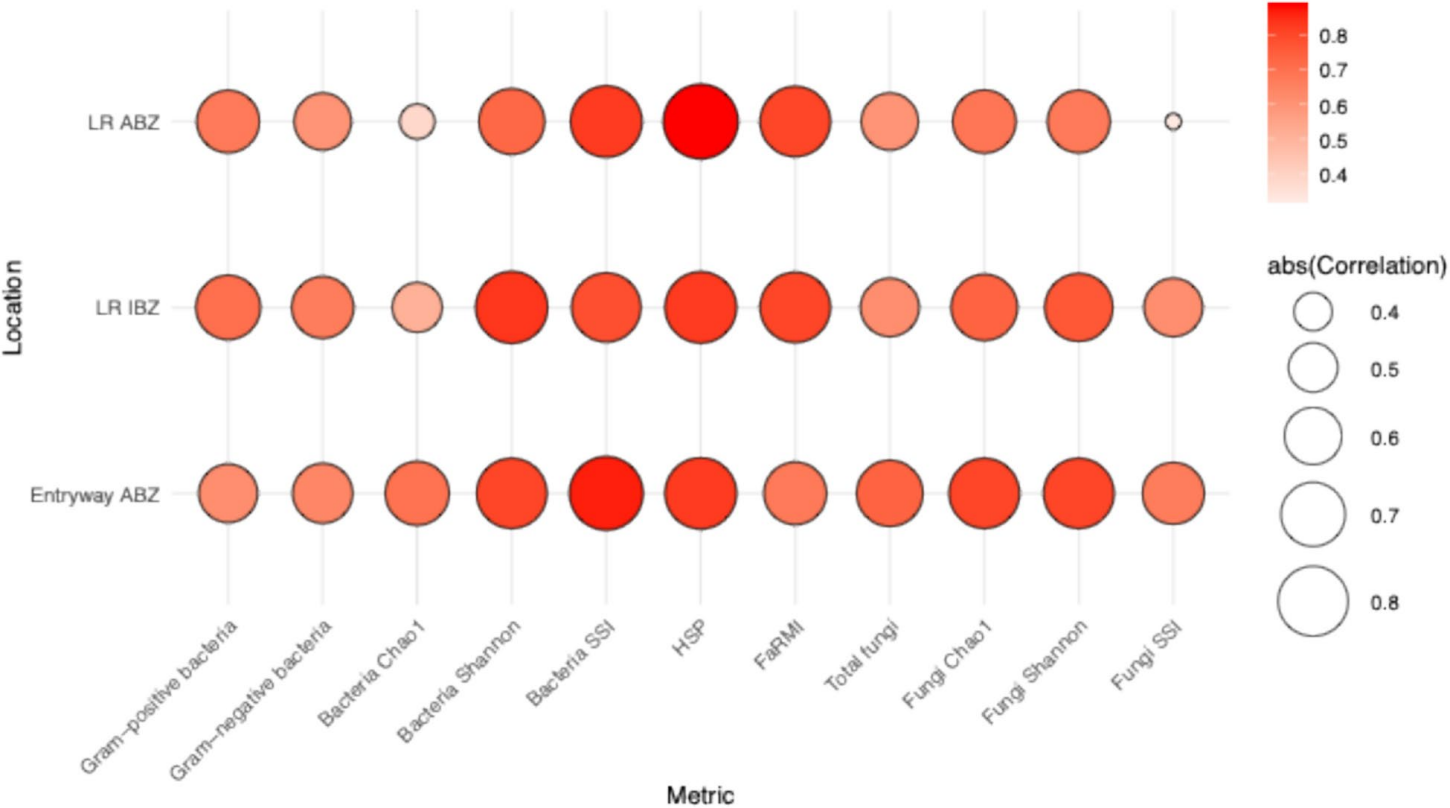
Spearman rank correlations between microbial metrics in entryway settled dust at infant breathing zone (IBZ; closest to the intervention rug) and the other settled dust sampling locations: entryway adult breathing zone (ABZ), living room (LR) IBZ, and LR ABZ. Results are shown for qPCR and alpha diversity measurements, soil source index (SSI), human source proxy (HSP), and the farm-home resembling microbiota index (FaRMI) metrics (*n* = 54–65 depending on the metric and sampling location). All correlations were significant at a *p* < 0.001, except for the fungi SSI *(p* = 0.018) and bacteria Chao1 (*p* = 0.002) at the LR ABZ location

Prior to soil seeding, there were no significant differences in community composition between settled dust samples (collected from both, IBZ and ABZ) collected in the entryways versus the living rooms (bacteria: *p* = 0.194; fungi: *p* = 0.063; PERMANOVA). Moreover, the qPCR measurements, alpha diversity, soil source proxy, human source proxy, and FaRMI, were mostly significantly and strongly correlated between the settled dust nearest to the intervention rug and the other sampling locations (*p* < 0.001; Fig. 3). Indoor and outdoor dust samples from all homes were compositionally dissimilar for both bacteria and fungi (*p* < 0.001; Supplemental Fig. 3; PERMANOVA). For outdoor samples, the residential environment (semi-urban vs. semi-rural) was a predictor of outdoor microbial community and was more important for determining bacterial assemblage than for fungal communities (bacteria: *p* < 0.001, *R*^2^ = 0.072, *F*-value = 20.064; fungi: *p* < 0.001, *R*^2^ = 0.057, *F*-value = 2.36; PERMANOVA). Correlations of microbial measurements between outdoor and indoor settled dust samples were consistently significant only for the qPCR measurements of Gram-negative bacteria (rho = 0.43–0.61 between outdoor and indoor settled dust in different locations), and in one comparison for total fungi (outdoor to indoor entryway IBZ; rho = 0.34) and FaRMI (outdoor to living room ABZ; rho = 0.32).

### Seeding soil characterization

Forest-collected soils had an average of 335 bacterial and 178 fungal ASVs per sample, and soil community composition remained stable over the course of the study, with no significant changes observed between the initial forest collection and the final seeding event (*p* = 0.154). At the phyla level, soils were dominated by Pseudomonadota (previously “Proteobacteria”; 45% of bacterial reads), Basidiomycota (55% of fungal reads), and Ascomycota (25% of fungal reads). *Bradyrhizobium*, a common soil associate, was the dominant bacterial genus (9% of reads), with an unidentified genus in the Acidobacteriae class (7.5%) and an unidentified genus in the Xanthobacteraceae family (7.5%) as the next most abundant genera (Fig. 1C). *Piloderma* was the most abundant fungal taxon recovered at the genus level of classification (25% of reads), followed by *Mortierella* (13%) and *Russula* (7%) (Fig. 1C; Supplemental Table 4 for the top 50 most abundant, genus level, bacterial and fungal taxa).

### Effects of soil-to rug interventions on the home microbiota

**Fig. 4:**
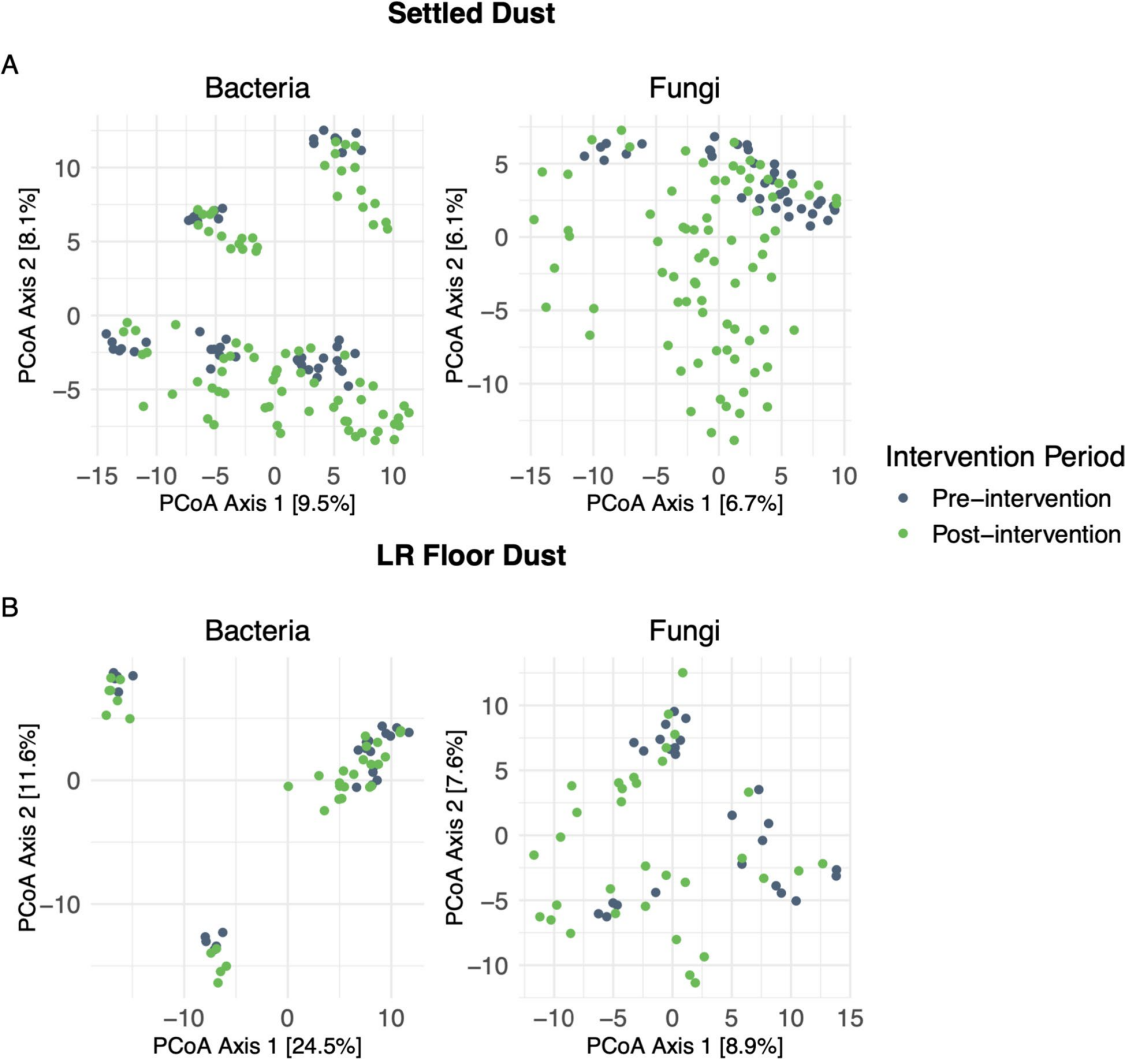
Microbial communities, pre- and post-intervention for settled and living room (LR) floor dust samples. Samples do not include those from control homes, and ordinations were calculated from ASV tables that were transformed with centered log-ratio and Aitchison distance. **A** Settled dust samples including all entryway and LR locations for both the IBZs and the ABZs (bacteria: *p* < 0.001; fungi: < 0.001). **B** LR floor dust was not significantly changed for bacteria (*p* = 0.097), but communities were significantly different pre- and post-intervention for fungi (*p* < 0.001). Two distinct clusters seen in the LR bacteria plot represent the two semi-rural homes that were included in the study (Homes 2 and 3)

Post-intervention, home-associated microbial communities became more similar to one another but remained distinct (*p* < 0.001 for all comparisons; Fig. 2 for settled dust and Supplemental Fig. 1 for floor dust samples). Home groupings were comparable to those seen pre-intervention for bacteria, less so for fungi. There was a significant (*p* < 0.001) shift in bacterial and fungal community structure post-intervention in both settled and LR floor dust samples (Fig. 4), except within LR floor bacteria (*p* = 0.097), across all intervention homes. Though there was a seasonal shift near the end of the study period in the control home, the effect was minor compared to changes observed from experimental homes following each intervention period.

**Fig. 5:**
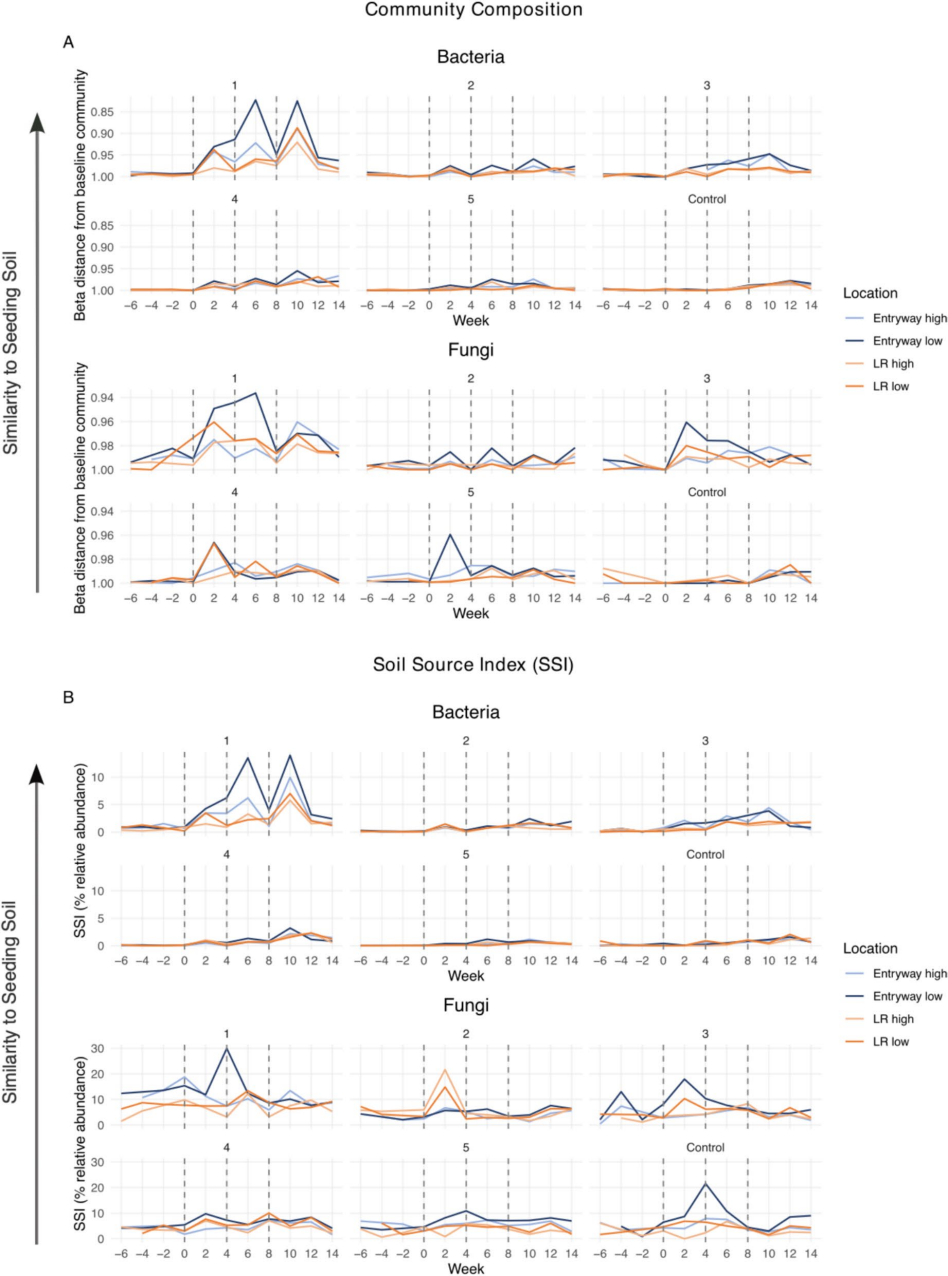
Similarity of settled dust to seeding soil, as measured by community composition (i.e., beta distances from seeding soil community) and a taxonomically informed soil source index (SSI) in the five intervention and one control homes. Dotted lines indicate intervention timepoints, for which samples were collected prior to the rug deployment. Higher peaks indicate greater similarity in home microbiome to the seeding soils. **A** Baseline community is the seeding soil that was used at each seeding event for each home, and beta distances were the distance values between baseline and intervention samples at each timepoint, based on Bray–Curtis dissimilarity. A decrease in beta distance indicates increased similarity in the community composition to the soil. **B** Represents a similar metric but was calculated as the sum relative abundance of the most abundant taxa found in the soil, rather than the whole community. Higher percent relative abundance indicates the samples were more similar to seeding soils

To consider the spatiotemporal dynamics associated with the soil seeding in airborne dust samples, we calculated the pairwise beta-diversity distances (Bray–Curtis) between the seeding soil and all settled dust samples at each timepoint. Compared to pre-seeding, the built environment microbiota became significantly similar to the soil two weeks post-seeding (PERMANOVA; bacteria: *p* < 0.001; fungi: *p* < 0.001, Fig. 5A), but the similarity decreased over time, from 2 weeks to four weeks post-seeding event. Further, the magnitude of response was dependent upon location in the home, with diminishing effects when moving both vertically and horizontally further away from the rug (Fig. 5A). In other words, the response was the strongest at the IBZ in the entryway and the weakest at the ABZ in the LR. Overall, the response to soil seeding was strongest in the home with the least number of occupants (Home 1; Fig. 5A), suggesting a potential swamping effect from occupant-associated microbes. We observed little additive effect of repeated seeding events, although the beta-diversity distances between the seeding soil and settled dust samples tended to be smallest after the third seeding event.

We, furthermore, performed statistical analysis on paired pre-post samples to study the effects of the soil on bacterial and fungal microbiota metrics measured from house dust. There were three pairs per home and per sample type, one at each of the three soil seeding events (weeks 0, 4, and 8). We compared the two weeks prior to soil-seeding versus two weeks after seeding time points (pre2w-post2w), as well as the two weeks prior to soil-seeding versus four weeks after seeding time points (pre2w-post4w) (Table 2 and Supplemental Tables 5 and 6 for intervention and control homes, respectively). Bacterial SSI was significantly increased across all dust sampling locations in the two weeks post-intervention (pre2w-post2w), i.e., in settled dust in the IBZs and ABZs in the entrance and living areas (*p* < 0.001), as well as in LR floor dust (*p* = 0.004) (Table 2; Fig. 5B). We observed a clear spatial dimension when tracking the soil signal within homes, with the highest post versus pre increase in bacterial SSI in the settled dust samples collected at the entrance IBZ (mean difference in relative abundance across intervention homes: 2.17%), followed by samples from entrance at the ABZ height (1.80%), living room IBZ (1.06%), and living room ABZ (0.94%) samples (Table 2). We also observed a significant, though smaller, increase in the LR floor dust samples (0.38%). Increases in bacterial SSI collected four weeks post-intervention (pre2w-post4w) were consistently smaller (0.49%–0.21%) than in the 2 weeks post samples, and only in a few cases were the pre2w-post4w comparisons significant, indicating that the soil signal in indoor dust diminishes over time. These results mirror the beta distances from seeding soil analysis (Bray–Curtis; Fig. 5A). The greatest influence of soil seeding on SSI was seen in home 1, with pre2w-post2w increases in entryway IBZ during the three individual seeding events ranging from 3.34% to 9.98% (Fig. 5B).

**Table 2:** Comparison of microbial parameters in paired pre- versus post-soil seeding samples. Three sample pairs were generated from each home, utilizing the three intervention time points, comparing the 2-week measurement preceding the seeding event to the 2 weeks after (pre2w-post2w), as well as the 2 weeks before seeding to the 4 weeks after (pre2w-post4w). *p *values were calculated with Student’s *t*-test or Wilcoxon Signed Rank test (in case of not normally distributed data) and are bolded to indicate statistical significance at *p* < 0.05. “Mean diff.” represents the mean of the differences between paired pre/post samples, where positive values indicate increases, and negative values decreases in post-seeding intervention compared to before. Measurement values are presented with three significant figures (except for values < 0.001)

		**Entrance IBZ (*n* = 13–14)**		**Entrance ABZ (*n* = 15)**		**LR IBZ (*n* = 14–15)**		**LR ABZ (*n* = 14–15)**		**LR floor dust (*n* = 13–14)**	
		Mean diff	*p*-value	Mean diff	*p*-value	Mean diff	*p*-value	Mean diff	*p*-value	Mean diff	*p*-value
**Bacteria SSI**	pre2w-post2w	2.17	**< *0.001***	1.80	**< *0.001***	1.06	**< *0.001***	0.94	**< *0.001***	0.38	***0.004***
	pre2w -post4w	0.46	*0.127*	0.40	*0.168*	0.49	***0.006***	0.35	***0.017***	0.21	*0.324*
**Bacteria Chao1**	pre2w -post2w	121	***0.036***	35.5	*0.412*	99.5	*0.073*	56.2	*0.133*	−0.92	*0.968*
	pre2w -post4w	64	*0.081*	86.5	*0.088*	108	***0.040***	74.7	***0.024***	9.15	*0.737*
**Bacteria Shannon**	pre2w -post2w	0.37	*0.052*	0.33	*0.065*	0.19	*0.295*	0.35	*0.086*	0.23	*0.305*
	pre2w -post4w	0.29	*0.094*	0.53	***0.023***	0.48	***0.031***	0.39	*0.082*	0.19	*0.171*
**HSP**	pre2w -post2w	−4.70	*0.071*	−2.14	*0.108*	0.56	*0.666*	−1.01	*0.450*	−3.12	*0.135*
	pre2w -post4w	−2.87	*0.183*	−2.95	***0.019***	−2.14	*0.145*	−3.17	*0.061*	0.09	*0.787*
**FaRMI**	pre2w -post2w	0.10	***0.013***	0.01	*0.208*	0.002	*0.974*	0.02	*0.497*	0.05	*0.135*
	pre2w -post4w	0.06	*0.121*	0.04	*0.166*	0.03	*0.476*	0.04	*0.389*	0.05	*0.282*
**Fungi SSI**	pre2w -post2w	−0.48	*0.952*	0.35	*0.714*	1.36	*0.286*	1.19	*0.903*	2.06	*0.096*
	pre-post4w	0.41	*0.340*	0.07	*0.359*	1.05	*0.078*	−0.10	*0.902*	0.79	*0.480*
**Fungi Chao1**	pre2w -post2w	11.2	*0.676*	49.6	*0.119*	69.0	***0.016***	25.7	*0.254*	−27.3	*0.604*
	pre-post4w	59.3	*0.077*	71.3	*0.072*	97.8	***0.005***	72.6	***0.017***	17.4	*0.637*
**Fungi Shannon**	pre2w -post2w	0.23	*0.196*	0.51	***0.017***	0.43	***0.032***	0.29	*0.247*	−0.10	*0.812*
	pre2w -post4w	0.27	*0.119*	0.43	*0.092*	0.52	***0.004***	0.42	*0.147*	−0.004	*0.992*

*SSI* soil source index, *Chao1* Chao1 richness estimate, *Shannon* Shannon diversity index, *HSP* humans source proxy, *FaRMI* farm-home resembling microbiota index, *IBZ* infant breathing zone, *ABZ* adult breathing zone, *LR* living room

Compared to the intervention homes, we observed only a marginal increase in bacterial SSI in the pre2w-post2w comparison in the entryway IBZ samples of the control home (0.08%) and small decreases in the other settled dust samples (−0.03 to −0.35%), while there was an increase in LR floor dust (0.94%) (Supplemental Table 6).

Additionally, we observed significant increases in bacterial Chao1 richness and FaRMI, as well as a borderline non-significant increase in Shannon diversity and a decrease in HSP for samples that were close to the intervention rug (entryway IBZ) for the two weeks post-intervention samples (Table 2). However, changes in the bacterial alpha-diversity measures were similar or even larger in the control home (Supplemental Table 6), which indicates that the effects may not be attributable to the intervention, even in the absence of statistical testing for the control samples (too low N). We did not identify consistent findings for the bacterial alpha-diversity, FaRMI, and HSP metrics in the house dust samples that were further away from the intervention rug or at 4 weeks post-intervention. Individual significant findings in these metrics and locations might be attributable to an outdoor influx of environmental microbes and the advance of spring, rather than to the soil intervention.

Unlike for the bacterial SSI, we did not find any indication for significant increases of the fungal SSI in the dust microbiota of the 2 weeks or 4 weeks post-soil seeding samples (Table 2; Fig. 5B). This seemingly opposes the earlier discussed finding from the fungal community analysis that showed increasing similarity to the soil post-intervention (Fig. 5A). There were significant increases in the Chao1 richness and Shannon diversity of house dust mycobiota, but those findings were not associated with proximity to the intervention rug (Table 2). Also, the response was typically stronger in the 4-week post compared to the 2-week post samples, and similar observations were made for the control home (Supplemental Table 6). Therefore, we consider that the magnitude of effect in fungal communities is less pronounced than that of bacterial communities, and rather, longitudinal changes in the house dust mycobiota seem to be more influenced by the onset of the spring than the intervention itself.

There were no obvious impacts of the soil seeding intervention on the quantitative bacterial and fungal levels in house dust (Supplemental Table 5).

### Observations concerning the microbiota metrics within home between dust samples and between homes

Complementing the main analysis around impacts of soil seeding interventions on indoor microbiota, we explored the temporal and spatial variability, as well as the within- and between-home variability of the bacterial and fungal richness and diversity metrics, qPCRs, HSP, and FaRMI, and observed partly striking differences. A detailed discussion of these observations is provided in the Supplement Text and in Supplemental Figs. 4–11.

## Discussion

We demonstrate here that a simple approach of seeding a few grams of forest soil onto rugs placed in the entrance area of a residential home can modify the airborne microbial communities toward that found in natural environments. Primarily, the intervention resulted in a relative enrichment of forest soil microbes in the indoor dust microbiota, reflected in an increase of a soil source bacteria index (SSI) and a shift in the community composition toward the seeding soil. We found that the effect was not limited to the immediate proximity of the intervention rugs, but the soil bacteria reached detectable increases in relative abundance in the living areas of the homes. Nevertheless, the soil bacteria signal decreased—as could be expected—with vertical and horizontal distance. Similarly, we observed a distinct temporal dimension, where the greatest difference from pre-intervention was observed in samples collected over the first 2 weeks after each seeding event, and the observed effect largely (but not entirely) diminished by the fourth week post-intervention. The soil seeding intervention did neither significantly affect bacterial and fungal quantities in house dust, nor consistently impact alpha-diversity and other microbiota metrics in the house dust samples. These results suggest that our intervention approach was specific to increasing the proportion of environmental, soil source bacteria, rather than causing major changes in richness or diversity of the house dust microbiota.

### Effects of the interventions on bacterial and fungal soil-source signatures in house dust microbiota

Bacterial and fungal house dust communities became more similar to the soil composition after each intervention (Fig. 5A). For bacteria, we also observed an increase in the relative abundance of genera characterized from the seeding soil, as indicated by the SSI, but that was not the case for fungi. The peaks of fungal SSI in house dust did not always correlate temporally with the soil seeding events. Notably, the fungal SSI was relatively high (5% to 15%) in most homes during the baseline period, before the introduction of the forest soil, unlike for bacteria, which had a low relative abundance of SSI-associated taxa before the intervention. This suggests that the fungal genera recovered from soil and contributing to the SSI were not exclusively associated with the seeding soil but were already part of the resident home mycobiota through other sources, such as outdoor air. It appears that spikes in relative abundances in the fungal SSI in house dust are partly caused by fungal strains that are taxonomically allocated to the fungal SSI genera, but are not, however, strains that are contained in the seeding soil. Thus, a higher percentage in fungal SSI is not necessarily reflected in the difference in beta-distance between dust samples and seeding soil. Supporting this, levels of fungal SSI in outdoor dust samples (medians over the study period across homes: 5.5%–16%; max. values over the study period across homes: 17%–38%) were clearly larger than the bacterial SSI levels in outdoor dust (medians: 3.6%–6.5%; max. values: 7.0%–19%). Irrespective of that, the beta-diversity shifts toward the soil source were clearly recognizable and correlated to the seeding events for fungi, at extents that were comparable to the effects on bacterial microbiota.

We did not observe any of the aforementioned, soil-associated effects in house dust bacteria from the non-intervention, control home, which was monitored alongside the experimental homes. There was a marginal increase in soil source bacteria toward the end of the study period; however, this increase neither matched the timing of the seeding events nor the extent that was observed with bacteria from experimental homes. We link this observation to the end of snow cover on the ground and the onset of spring and summer, which leads to an increase of outdoor environmental microbes indoors through outdoor-indoor transfers via air and clothing, especially shoes. The fungal beta-diversity in the control home only marginally shifted toward the seeding soil at the end of the observation period, similar to what we found for bacteria. Fungal soil source taxa were abundant in the control home at baseline and throughout the study period, as seen also for the intervention homes.

### Implications of temporospatial characteristics of the interventions

Our observations concerning the spatial and temporal dynamics of the soil-on-carpet seeding interventions are informative to future efforts that will attempt to use similar methods to study potential changes in the human microbiome, immunological markers, or immunological health outcomes. Our findings suggest that the impact of introducing soil in low quantities into homes on the airborne dust microbiota are: specific, in increasing the proportion of environmental, soil microbes in the indoor microbiota rather than impacting the richness and diversity of indoor microbiota more generally; local, in that the strongest modifications are observed close to the soil source; and temporally limited, with a clear decline of the intervention effects within four weeks post-soil seeding. We performed three subsequent forest soil seeding events in each intervention home, four weeks apart, and thus expected some cumulative enrichment of soil bacteria in the dust microbiota over time. However, while we could observe a gradual shift in the bacterial community structure toward the soil communities, this trend was subtle.

These characteristics and the dynamic nature of the observed effects suggest that exposure rate is local and reversible, rather than spatially uncontrolled and permanent. We observed the greatest changes in airborne dust microbiota in the IBZ close to the intervention carpet, which is an important consideration for the alteration and/or measurement of health outcomes, as this or a similar approach would likely be used to target infants and small children. The first 2 years of life, in addition to the last trimester of pregnancy, are often referred to as “the window of opportunity,” during which exposure to diverse, environmental microbes has the greatest potential to affect immune system development and human microbiome maturation in health-promoting ways [8, 57]. If a non-pharmaceutical approach toward promoting beneficial microbial exposure via modification of the indoor living environment during early life stages is the goal, seeding of environmental microbes onto a blanket, an infant play mat, or a baby cot could be alternative strategies. Obviously, such approaches will need to consider the fact that there can be pathogens or otherwise health hazardous microbes in environmental matrices and control the associated risks. Our observation of the temporal degradation of such intervention would imply the need for repeated introduction to increase and maintain exposure, but at the same time adds control over the treatment approach.

### Environmental interventions to promote microbial encounter

Recent research efforts aimed at recreating historic biodiverse interactions in our daily lives have shown that environmental interventions can diversify human microbiota, but adequately powered studies with long-term follow-up on measurable health endpoints are lacking [21]. Previous research has used a variety of different approaches such as outdoor preschool programs [58], the construction of structures that maintain indoor plants (such as green walls in offices [59]), or converting outdoor daycare spaces in ways that resemble the forest floor ecosystem [60]. However, these efforts can be expensive, logistically difficult to implement and maintain, and might not modify early-life exposures during the optimal window of opportunity. Thus, their potential scope of use is limited. In this context, the residential home is an important point of contact, because this is the location where the majority of expecting mothers and their newborn children will spend most of their time.

Living architecture and indoor ornamental plants have been recognized as sources of diverse indoor microbiota, but associations with immunological health outcomes are only speculative at this stage, due to the absence of dedicated epidemiological research [61, 62]. One early study in a controlled, cleaned, non-occupied chamber environment, in which a plant was placed for several months, found increases in the abundance of archaea, bacteria, and fungi on the chamber surfaces [63]. While this study demonstrated the mechanism of plant microbiota-to-built environment transfer, the findings are not directly translatable to occupied indoor environments with a multitude of other microbial sources. Addressing the latter, a recent study in US offices found no significant effects of the presence of plants on alpha and beta diversity or the abundance of mycobiota, with only a small fraction of highly abundant plant soil ASVs detectable in the dust samples (0.6 ± 1.5%) [64]. In contrast, a study in Belgian residences found significant increases in estimated bacterial and fungal richness (Chao1) when comparing homes with no plants to homes with three or more plants [65]. This research also investigated the effects of different types of residential green space surrounding the homes on house dust microbiota [3] and reported increases in indoor bacterial and fungal Shannon diversity (but not richness) with more nearby residential greenery. Interestingly, indoor bacterial and fungal communities were differentially impacted by the outdoor green characteristics in this and one earlier study [3, 66]. Beyond the simple fact that “outdoor green” is not readily available in desired quantities and qualities in all urban environments, such research indicates the challenges associated with harnessing outdoor or indoor environmental characteristics to modify indoor microbiota in predictable ways.

### The relevance of home characteristics

We found consistent spatial and temporal variation patterns among the study homes, despite differences in home characteristics (Table 1), suggesting this intervention method would be applied to homes of different types, years of construction, location, and use patterns. Nevertheless, home characteristics (including occupant demographics) were determinants of the magnitude of change in community composition following seeding interventions. Study home 1 was different from other study homes in that the soil seeding altered the bacterial community composition to a greater extent when compared to the other homes. Home 1 differed from the others in various aspects, all of which are well-known determinants of house dust microbial assemblies [67, 68], including that it was the only apartment (as opposed to detached and semi-detached study homes), was located on the eighth floor, had the lowest occupancy (one adult and one child), was the newest built and smallest space, and was fully mechanically ventilated. The homes in our study were comparable in frequency at which the rug was walked over, most reporting 20 or more passages over the carpet daily, except for home 1. Though we saw the greatest response in home 1, there was comparable, but less, contact with the rug (10–20 passages over the rug daily), suggesting the other defining home characteristics and occupant demographics were more important than the rate of contact. It is reasonable to assume that intervention impacts would be more pronounced in an indoor space where other competing microbial sources are less strong (e.g., fewer people living in the home), and our observations from home 1 corroborate such an assumption, though obviously limited by the minimal number of replications. This could and should be considered with respect to best practices for implementing and tailoring intervention strategies based on the demographic characteristics of the residence. For example, the use of greater quantities of soil, higher frequency of repeated dosing, or more rugs in homes with additional microbial sources. Then again, the basic motivation of our intervention lies in efforts to counteract depletion of environmental microbial contacts associated with high levels of urbanization. It appears that an environmental microbiota transfer, as suggested here, would be most effective in residences where this would be most necessary with respect to supporting beneficial microbial interactions.

### Potential health dimension of this intervention

Here, we used forest soil as the matrix to facilitate environmental microbiota transfers into urban homes. This choice was less based on potential health effects associated with exposure to bacteria or fungi in pristine forest soil. Rather, this is a proof of concept study for using forest soil to alter microbiomes in ways that could potentially mimic the indoor exposures observed in rural or farming homes [69]. Nevertheless, previous research has suggested health relevance and benefits of contact with soil microbes [24, 69–71]. Some of the bacterial and fungal genera identified among the dominant taxa in our forest soil samples, such as *Mycobacterium*, *Mortierella*, and *Russula*, have been shown to have human health-promoting properties [72–74]. Additionally, we observed an increase in the Farmhouse Resembling Microbiota Index (FaRMI) community members, though these findings were restricted to airborne dust samples collected close to the source. The FaRMI quantifies a microbial composition associated with asthma protection, characterized by the similarity of house dust microbiota to that found in farm homes [19]. An increase in FaRMI is roughly representative of a shift toward bacterial communities dominated by outdoor-origin taxa over human-associated taxa, particularly those including potential respiratory pathogens. In this study, the interventions were associated with variable changes in FaRMI, with a mean increase of 0.1 across all homes. Based on our earlier data [19], this would be associated with an odds ratio (OR) of 0.8 for asthma prior to school age in a child growing up in such a home, compared to a home without the intervention. The largest mean increase in FaRMI observed in a single home was 0.19, which would correspond to an OR of 0.7. Notably, this represents an effect size similar to the protection associated with growing up on farms [75]. While some uncertainty remains regarding how much of the FaRMI increase can truly be attributed to the intervention, the results are encouraging and suggest potential for a comparable approach—especially if further developed and targeted to low-FaRMI homes occupied by families at risk for asthma.

### Strengths and weaknesses

This study was tailored to observe spatiotemporal impacts of repeated soil-seeding interventions on the house dust microbiota in varied, occupied, real-life residential settings, following a longitudinal research approach. As such, the strengths of the study include dedicated sampling efforts, collecting house dust repeatedly over 22 weeks at different locations and different heights in the study homes (matched with repeated outdoor sampling); the inclusion of both quantitative and qualitative measures of bacterial and fungal house dust microbiota; and, importantly, the research design with its standardized, repeated soil-seeding interventions and longitudinal dimension. The depth in sampling and measurements and implementation of repeated interventions was logistically challenging and came at the expense of needing to limit the number of study and especially control homes. The study design with a low number of homes and partly overlapping variables did not allow for a detailed characterization of determinants of the indoor microbiota, which was, however, also not the aim of this study. The longitudinal observation period of five months resulted in that outdoor environmental conditions, including climate and microbiota, changed drastically when moving from winter through spring into early summer, which also impacted the indoor microbiota characteristics. The combination of these aspects complicated some of the statistical analyses, for example, when trying to parse effects of the soil-seeding intervention from the onset of spring in more variable microbiota measures (e.g., taxa richness and diversity). We were also limited in our ability to interpret our findings in subsets of homes with specific characteristics in terms of their microbial determinants.

All study homes were in semi-urban and semi-rural areas in Finland and are representative of the Finnish building stock and specific occupant living habits. As such, the wider applicability of the study findings remains to be confirmed in future research. This study did, however, include a large variety of Finnish homes in terms of number of occupants, pets, residence type, and building characteristics, and our results indicate that such or similar indoor microbiota interventions could be successfully implemented in various residence types and especially in highly urbanized settings. As a proof-of-principle investigation, we consider that our study provides novel and highly relevant information to future research efforts attempting house dust microbiota modifications in efforts to provide health-promoting indoor environments.

## Conclusions

We demonstrate here that environmental microbiota transfer into urban homes, via the simple intervention of placing a forest soil-seeded rug indoors, allows for a feasible means of modifying the airborne indoor microbiota. There are spatial and temporal dynamics linked to such interventions, and the effects appear to be strongest in residences where other competing microbiota sources are relatively weak. While the effects of the intervention on the indoor microbiota diversity and composition are variable between homes, the approach as such appears promising, specifically in highly urbanized settings. Aspects of dosage and type of environmental microbiota to reach health-promoting quantities and qualities useful to asthma prevention require further study.

## Supplementary Information

Additional file 1. Supplemental text, Supplemental figures, and Supplemental Tables.

Additional file 2. Floor dust dataset.

Additional file 3. Settled dust dataset.

## Data Availability

Raw sequence files and metadata are publicly available in the European Nucleotide Archive (ENA; Accession PRJEB98135). Additionally, all R code used for 16S and ITS analyses is available on GitHub at https://github.com/soil_in_homes.

## References

[1] Parajuli A, Grönroos M, Siter N, Puhakka R, Vari HK, Roslund MI, et al. Urbanization reduces transfer of diverse environmental microbiota indoors. Front Microbiol. 2018;9:84. 29467728 10.3389/fmicb.2018.00084 PMC5808279

[2] Leppänen HK, Täubel M, Jayaprakash B, Vepsäläinen A, Pasanen P, Hyvärinen A. Quantitative assessment of microbes from samples of indoor air and dust. J Expo Sci Environ Epidemiol. 2018;28:231–41. 28975927 10.1038/jes.2017.24

[3] Dockx Y, Täubel M, Bijnens EM, Witters K, Valkonen M, Jayaprakash B, et al. Residential green space can shape the indoor microbial environment. Environ Res. 2021;201:111543. 10.1016/j.envres.2021.111543 34157273

[4] Haahtela T. A biodiversity hypothesis. Allergy. 2019;74:1445–56. 30835837 10.1111/all.13763

[5] Mills JG, Weinstein P, Gellie NJC, Weyrich LS, Lowe AJ, Breed MF. Urban habitat restoration provides a human health benefit through microbiome rewilding: the Microbiome Rewilding Hypothesis: urban microbiome rewilding restores human health. Restor Ecol. 2017;25:866–72.

[6] Mills JG, Brookes JD, Gellie NJC, Liddicoat C, Lowe AJ, Sydnor HR, et al. Relating urban biodiversity to human health with the “holobiont” concept. Front Microbiol. 2019;10:550. 30972043 10.3389/fmicb.2019.00550 PMC6444116

[7] Strachan DP. Hay fever, hygiene, and household size. BMJ. 1989;299:1259–60. 2513902 10.1136/bmj.299.6710.1259 PMC1838109

[8] Kloepfer KM, McCauley KE, Kirjavainen PV. The microbiome as a gateway to prevention of allergic disease development. J Allergy Clin Immunol Pract. 2022;10:2195–204. 35718258 10.1016/j.jaip.2022.05.033

[9] Lehtimäki J, Sinkko H, Hielm-Björkman A, Salmela E, Tiira K, Laatikainen T, et al. Skin microbiota and allergic symptoms associate with exposure to environmental microbes. Proc Natl Acad Sci U S A. 2018;115:4897–902. 29686089 10.1073/pnas.1719785115 PMC5948976

[10] Hanski I, von Hertzen L, Fyhrquist N, Koskinen K, Torppa K, Laatikainen T, et al. Environmental biodiversity, human microbiota, and allergy are interrelated. Proc Natl Acad Sci U S A. 2012;109:8334–9. 22566627 10.1073/pnas.1205624109 PMC3361383

[11] Ruokolainen L, von Hertzen L, Fyhrquist N, Laatikainen T, Lehtomäki J, Auvinen P, et al. Green areas around homes reduce atopic sensitization in children. Allergy. 2015;70:195–202. 25388016 10.1111/all.12545 PMC4303942

[12] Zhang Y-D, Fan S-J, Zhang Z, Li J-X, Liu X-X, Hu L-X, et al. Association between residential greenness and human microbiota: evidence from multiple countries. Environ Health Perspect. 2023;131:87010. 37585351 10.1289/EHP12186 PMC10431502

[13] Asri AK, Liu T, Tsai H-J, Lee H-Y, Pan W-C, Wu C-D, et al. Residential greenness and air pollution’s association with nasal microbiota among asthmatic children. Environ Res. 2023;219:115095. 36535395 10.1016/j.envres.2022.115095

[14] Depner M, Taft DH, Kirjavainen P, Kalanetra K, Karvonen A, Peschel S, et al. Maturation of the gut microbiome during the first year of life contributes to the protective farm effect on childhood asthma. Nat Med. 2020;26:1766–75. 10.1038/s41591-020-1095-x 33139948

[15] Lehtimäki J, Thorsen J, Rasmussen MA, Hjelmsø M, Shah S, Mortensen MS, et al. Urbanized microbiota in infants, immune constitution, and later risk of atopic diseases. J Allergy Clin Immunol. 2021;148:234–43. 33338536 10.1016/j.jaci.2020.12.621

[16] Long A, Kleiner A, Looney RJ. Immune dysregulation. J Allergy Clin Immunol. 2023;151:70–80. 36608984 10.1016/j.jaci.2022.11.001

[17] Asthma. [cited 2025 Mar 5]. Available from: https://www.who.int/news-room/fact-sheets/detail/asthma?utm_source=chatgpt.com

[18] Haahtela T, Lindholm H, Björkstén F, Koskenvuo K, Laitinen LA. Prevalence of asthma in Finnish young men. BMJ. 1990;301:266–8. 2390620 10.1136/bmj.301.6746.266 PMC1663451

[19] Kirjavainen PV, Karvonen AM, Adams RI, Täubel M, Roponen M, Tuoresmäki P, et al. Farm-like indoor microbiota in non-farm homes protects children from asthma development. Nat Med. 2019;25:1089–95. 31209334 10.1038/s41591-019-0469-4 PMC7617062

[20] Karvonen AM, Kirjavainen PV, Täubel M, Jayaprakash B, Adams RI, Sordillo JE, et al. Indoor bacterial microbiota and development of asthma by 10.5 years of age. J Allergy Clin Immunol. 2019;144:1402–10. 31415782 10.1016/j.jaci.2019.07.035

[21] Tischer C, Kirjavainen P, Matterne U, Tempes J, Willeke K, Keil T, et al. Interplay between natural environment, human microbiota and immune system: a scoping review of interventions and future perspectives towards allergy prevention. Sci Total Environ. 2022;821:153422. 35090907 10.1016/j.scitotenv.2022.153422

[22] Stein MM, Hrusch CL, Gozdz J, Igartua C, Pivniouk V, Murray SE, et al. Innate immunity and asthma risk in Amish and Hutterite farm children. N Engl J Med. 2016;375:411–21. 27518660 10.1056/NEJMoa1508749 PMC5137793

[23] Honeker LK, Sharma A, Gozdz J, Theriault B, Patil K, Gimenes JA Jr, et al. Gut microbiota from Amish but not Hutterite children protect germ-free mice from experimental asthma. D92 THE MICROBIOME AND LUNG DISEASE. American Thoracic Society; 2019. Available from: 10.1164/ajrccm-conference.2019.199.1_meetingabstracts.a7022

[24] Ottman N, Ruokolainen L, Suomalainen A, Sinkko H, Karisola P, Lehtimäki J, et al. Soil exposure modifies the gut microbiota and supports immune tolerance in a mouse model. J Allergy Clin Immunol. 2019;143:1198-1206.e12. 30097187 10.1016/j.jaci.2018.06.024

[25] Shorter CL. Fungi in New Zealand homes: measurement, aerosolisation & association with children’s health. University of Otago; 2013 [cited 2025 Feb 26]. Available from: https://ourarchive.otago.ac.nz/esploro/outputs/doctoral/Fungi-in-New-Zealand-homes-Measurement/9926479519301891

[26] Causer SM, Piper C, Shorter CL, Lewis RD. Replicating the cross-sectional distribution of house-dust mite allergen found in carpet. J Text Inst. 2010;101:69–75.

[27] Adams RI, Tian Y, Taylor JW, Bruns TD, Hyvärinen A, Täubel M. Passive dust collectors for assessing airborne microbial material. Microbiome. 2015;3:46. 26434807 10.1186/s40168-015-0112-7 PMC4593205

[28] Haugland RA, Siefring S, Lavender J, Varma M. Influences of sample interference and interference controls on quantification of enterococci fecal indicator bacteria in surface water samples by the qPCR method. Water Res. 2012;46:5989–6001. 22981586 10.1016/j.watres.2012.08.017

[29] Kärkkäinen PM, Valkonen M, Hyvärinen A, Nevalainen A, Rintala H. Determination of bacterial load in house dust using qPCR, chemical markers and culture. J Environ Monit. 2010;12:759–68. 20445866 10.1039/b917937b

[30] Meklin T, Haugland RA, Reponen T, Varma M, Lummus Z, Bernstein D, et al. Quantitative PCR analysis of house dust can reveal abnormal mold conditions. J Environ Monit. 2004;6:615–20. 15237292 10.1039/b400250d PMC2233939

[31] Caporaso JG, Lauber CL, Walters WA, Berg-Lyons D, Lozupone CA, Turnbaugh PJ, et al. Global patterns of 16S rRNA diversity at a depth of millions of sequences per sample. Proc Natl Acad Sci U S A. 2011;108(Suppl 1):4516–22. 20534432 10.1073/pnas.1000080107 PMC3063599

[32] Smith DP, Peay KG. Sequence depth, not PCR replication, improves ecological inference from next generation DNA sequencing. PLoS One. 2014;9:e90234. 24587293 10.1371/journal.pone.0090234 PMC3938664

[33] Martin M. Cutadapt removes adapter sequences from high-throughput sequencing reads. EMBnetjournal. 2011;17:10–2.

[34] Weinstein MM, Prem A, Jin M, Tang S, Bhasin JM. FIGARO: an efficient and objective tool for optimizing microbiome rRNA gene trimming parameters. bioRxiv. 2019 [cited 2023 Apr 10]. p. 610394. Available from: https://www.biorxiv.org/content/10.1101/610394v1.abstract

[35] Callahan BJ, McMurdie PJ, Rosen MJ, Han AW, Johnson AJA, Holmes SP. DADA2: high-resolution sample inference from Illumina amplicon data. Nat Methods. 2016;13:581–3. 27214047 10.1038/nmeth.3869 PMC4927377

[36] Quast C, Pruesse E, Yilmaz P, Gerken J, Schweer T, Yarza P, et al. The SILVA ribosomal RNA gene database project: improved data processing and web-based tools. Nucleic Acids Res. 2013;41:D590–6. 10.1093/nar/gks1219 PMC3531112 23193283

[37] Nilsson RH, Larsson K-H, Taylor AFS, Bengtsson-Palme J, Jeppesen TS, Schigel D, et al. The UNITE database for molecular identification of fungi: handling dark taxa and parallel taxonomic classifications. Nucleic Acids Res. 2019;47:D259–64. 30371820 10.1093/nar/gky1022 PMC6324048

[38] Davis NM, Proctor DM, Holmes SP, Relman DA, Callahan BJ. Simple statistical identification and removal of contaminant sequences in marker-gene and metagenomics data. Microbiome. 2018;6:226. 30558668 10.1186/s40168-018-0605-2 PMC6298009

[39] R Core Team. _R: a language and environment for statistical computing_ . 2023. Available from: https://www.R-project.org/

[40] McMurdie PJ, Holmes S. Phyloseq: an R package for reproducible interactive analysis and graphics of microbiome census data. PLoS One. 2013;8:e61217. 23630581 10.1371/journal.pone.0061217 PMC3632530

[41] Oksanen J, Blanchet FG, Friendly M, Kindt R, Legendre P, McGlinn D, et al. vegan: community ecology package (2.6-4). 2022; Available from: https://scholar.google.com/citations?view_op=view_citation&hl=en&citation_for_view=2WBRFVIAAAAJ:QsaTk4IG4EwC

[42] Wickham H, François R, Henry L, Müller K, Vaughan D. dplyr: a grammar of data manipulation. 2023. Available from: https://dplyr.tidyverse.org

[43] Wickham H. Ggplot2. Wiley Interdiscip Rev Comput Stat. 2011;3:180–5.

[44] Wickham H. ggplot2: elegant graphics for data analysis. Springer-Verlag New York; 2016. Available from: https://ggplot2.tidyverse.org

[45] Kassambara A. ggpubr: ‘ggplot2’ based publication ready plots. R package version 0.6.0. 2023.

[46] Arnold JB. ggthemes: extra themes, scales and geoms for ggplot2. R package version 5.1. 0. 2024.

[47] Pedersen TL. patchwork: the composer of plots. 2024. Available from: https://CRAN.R-project.org/package=patchwork

[48] Wilcoxin F. Probability tables for individual comparisons by ranking methods. Biometrics. 1947;3:119–22. 18903631

[49] Kruskal WH, Wallis WA. Use of ranks in one-criterion variance analysis. J Am Stat Assoc. 1952;47:583.

[50] Aitchison J. The statistical analysis of compositional data. J R Stat Soc Ser B Stat Methodol. 1982;44:139–60.

[51] Gloor GB, Macklaim JM, Pawlowsky-Glahn V, Egozcue JJ. Microbiome datasets are compositional: and this is not optional. Front Microbiol. 2017;8:2224. 29187837 10.3389/fmicb.2017.02224 PMC5695134

[52] Martino C, Morton JT, Marotz CA, Thompson LR, Tripathi A, Knight R, et al. A novel sparse compositional technique reveals microbial perturbations. mSystems. 2019. 10.1128/mSystems.00016-19. 30801021 10.1128/mSystems.00016-19 PMC6372836

[53] Kodera SM, Sharma A, Martino C, Dsouza M, Grippo M, Lutz HL, et al. Microbiome response in an urban river system is dominated by seasonality over wastewater treatment upgrades. Environ Microbiome. 2023;18:10. 36805022 10.1186/s40793-023-00470-4 PMC9938989

[54] Hospodsky D, Qian J, Nazaroff WW, Yamamoto N, Bibby K, Rismani-Yazdi H, et al. Human occupancy as a source of indoor airborne bacteria. PLoS One. 2012;7:e34867. 22529946 10.1371/journal.pone.0034867 PMC3329548

[55] Haugland RA, Vesper SJ. Method of identifying and quantifying specific fungi and bacteria. US: Patent 6387652 B1; 2002.

[56] Haugland RA, Varma M, Wymer LJ, Vesper SJ. Quantitative PCR analysis of selected Aspergillus, Penicillium and Paecilomyces species. Syst Appl Microbiol. 2004;27:198–210. 15046309 10.1078/072320204322881826

[57] von Mutius E, Vercelli D. Farm living: effects on childhood asthma and allergy. Nat Rev Immunol. 2010;10:861–8. 21060319 10.1038/nri2871

[58] Sobko T, Liang S, Cheng WHG, Tun HM. Impact of outdoor nature-related activities on gut microbiota, fecal serotonin, and perceived stress in preschool children: the Play&Grow randomized controlled trial. Sci Rep. 2020;10:21993. 33319792 10.1038/s41598-020-78642-2 PMC7738543

[59] Soininen L, Roslund M, Nurminen N, Puhakka R, Laitinen O, Hyöty H, et al. Indoor green wall affects health-associated commensal skin microbiota and enhances immune regulation: a randomized trial among urban office workers. Sci Rep. 2022. 10.1038/s41598-022-10432-4. 35444249 10.1038/s41598-022-10432-4 PMC9021224

[60] Roslund MI, Puhakka R, Grönroos M, Nurminen N, Oikarinen S, Gazali AM, et al. Biodiversity intervention enhances immune regulation and health-associated commensal microbiota among daycare children. Sci Adv. 2020. 10.1126/sciadv.aba2578. 33055153 10.1126/sciadv.aba2578 PMC7556828

[61] Prussin AJ 2nd, Marr LC. Sources of airborne microorganisms in the built environment. Microbiome. 2015;3:78. 26694197 10.1186/s40168-015-0144-z PMC4688924

[62] Gilbert JA, van der Hartmann EM. The indoors microbiome and human health. Nat Rev Microbiol. 2024;22:742–55. 39030408 10.1038/s41579-024-01077-3

[63] Mahnert A, Moissl-Eichinger C, Berg G. Microbiome interplay: plants alter microbial abundance and diversity within the built environment. Front Microbiol. 2015 [cited 2022 May 11];6. Available from: https://www.frontiersin.org/article/10.3389/fmicb.2015.00887 10.3389/fmicb.2015.00887 PMC4552223 26379656

[64] Leslie A, Chowdhury MR, Täubel M, Hegarty B. Indoor potted plants have little effect on office dust fungal communities. Indoor Environ. 2025;2:100092.

[65] Dockx Y, Täubel M, Bijnens EM, Witters K, Valkonen M, Jayaprakash B, et al. Indoor green can modify the indoor dust microbial communities. Indoor Air. 2022;32:e13011. 35347789 10.1111/ina.13011

[66] Weikl F, Tischer C, Probst AJ, Heinrich J, Markevych I, Jochner S, et al. Fungal and bacterial communities in indoor dust follow different environmental determinants. PLoS One. 2016;11:e0154131. 27100967 10.1371/journal.pone.0154131 PMC4839684

[67] Rintala H, Pitkäranta M, Täubel M. Microbial communities associated with house dust. Adv Appl Microbiol. 2012;78:75–120. 22305094 10.1016/B978-0-12-394805-2.00004-X

[68] National Academy of Sciences, Medicine, on Engineering D, Sciences P, Division M, on Earth D, et al. Microbiomes of the built environment: a research agenda for indoor microbiology, human health, and buildings. 2017; Available from: https://nap.nationalacademies.org/23647 29035489

[69] Banerjee S, van der Heijden MGA. Soil microbiomes and one health. Nat Rev Microbiol. 2023;21:6–20. 35999468 10.1038/s41579-022-00779-w

[70] Ruokolainen L, Fyhrquist N, Haahtela T. The rich and the poor: environmental biodiversity protecting from allergy. Curr Opin Allergy Clin Immunol. 2016;16:421–6. 27490122 10.1097/ACI.0000000000000304

[71] von Hertzen L, Haahtela T. Disconnection of man and the soil: reason for the asthma and atopy epidemic? J Allergy Clin Immunol. 2006;117:334–44. 16461134 10.1016/j.jaci.2005.11.013

[72] Dyal SD, Narine SS. Implications for the use of *Mortierella* fungi in the industrial production of essential fatty acids. Food Res Int. 2005;38:445–67.

[73] Oliveira RB, Robl D, Ienczak JL. Potential of Mortierellaceae for polyunsaturated fatty acids production: mini review. Biotechnol Lett. 2023;45:741–59. 37148344 10.1007/s10529-023-03381-z

[74] Cheng Y, Gan J, Yan B, Wang P, Wu H, Huang C. Polysaccharides from *Russula*: a review on extraction, purification, and bioactivities. Front Nutr. 2024;11:1406817. 38746936 10.3389/fnut.2024.1406817 PMC11091342

[75] Pechlivanis S, von Mutius E. Effect of farming on asthma. Acta Med Acad. 2020;49:144–55. 33189120 10.5644/ama2006-124.293

