# Supplementary material for: Environmental microbiota transfer from forest soil into urban homes: a proof-of-principle study
Source: Microbiome. 2026 Mar 25;14:95. doi: 10.1186/s40168-026-02352-6 (PMC13019970; doi:10.1186/s40168-026-02352-6)
Supplement: Supplementary file 2 — Additional file 1. Supplemental text, Supplemental figures, and Supplemental Tables. [file 40168_2026_2352_MOESM1_ESM.docx]

**SUPPLEMENT**

**Environmental microbiota transfer from forest soil into urban homes: a proof-of-principle study.**

Martin Täubel^1,2*†^, Megan S. Hill^3,4†^, Sarah Allard^3,4,5^, Jack A. Gilbert^3,4,5^, Maria Valkonen^1^, Anne M. Karvonen^1^, Asko Vepsäläinen^1^, Juha Pekkanen^1,6^, Pirkka V. Kirjavainen^1,7^

^1^Lifestyles and Living Environments Unit, Department Public Health, Finnish Institute for Health and Welfare, Kuopio, Finland

^2^Department of Civil Engineering, Aalto University, Espoo, Finland

^3^Department of Pediatrics, University of California San Diego School of Medicine, La Jolla, CA, USA

^4^Center for Marine Biotechnology and Biomedicine, Scripps Institution of Oceanography, University of California, San Diego, La Jolla, CA, USA

^5^Soil Health Center, Scripps Institution of Oceanography, University of California, San Diego, La Jolla, CA, USA

^6^Department of Public Health, University of Helsinki, Helsinki, Finland

^7^Institute of Public Health and Clinical Nutrition, University of Eastern Finland, Kuopio, Finland.

^†^Equal contribution

^*^Corresponding author: *martin.taubel@thl.fi*

**Supplement text.**

***Observations concerning the microbiota metrics within home between dust samples and between homes.***

For the bacterial and fungal taxa richness (Chao1) in particular, but also for Shannon diversity, we observed large variability in settled dust samples within the homes over time, with comparably rather subtle differences between homes (Supplemental Figure 4). An exception was Home 5, which was characterized by high fungal richness but low Shannon diversity. Bacterial and fungal diversity and the Farm-Home Resembling Microbiota Index (FaRMI; Supplemental Figure 5) tended to increase in the study homes over time, more so than taxa richness. This observation is likely due to the advent of spring and the loss of snow cover on the ground, resulting in a general increase in environmental microbiota in the study homes. In line with that, we observed an opposite trend, i.e. a decrease in the proportion of human sourced bacteria (HSP) in house dust over the course of the study period (Supplemental Figure 5).

Bacterial and fungal richness was mostly and rather consistently higher in the settled dust samples collected close to floor level, compared to the samples collected in the adult breathing zone, and tended to be higher in the entrance area compared to the living room (Supplemental Figures 6 and 7). Fungal taxa richness was consistently higher in vacuumed living room floor dust compared to living room settled dust, while this was not the case for bacterial taxa richness. Bacterial Shannon diversity was mostly higher in the infant compared to the adult breathing zone, but was lowest in the floor dust samples (except for Home 5; Supplemental Figure 8). The relations between infant and adult breathing zone settled dust were similar for the fungal Shannon diversity, but unlike for bacteria, in most cases fungal diversity was highest in the floor dust samples (Supplemental Figure 9). We found striking differences in the percentage of human source proxy (HSP) between homes, ranging from means of below 10% to over 40%, with the lowest HSP values observed in the homes with multiple dogs (Homes 2 and 3; Supplemental Figure 10). Unlike for other metrics, there were no consistent differences in this bacterial metric between house dust sample types. Similarly, FaRMI was markedly different between the study homes, with no consistent difference between the measurements in the different house dust samples (Supplemental Figure 11).

**Supplemental Figures**


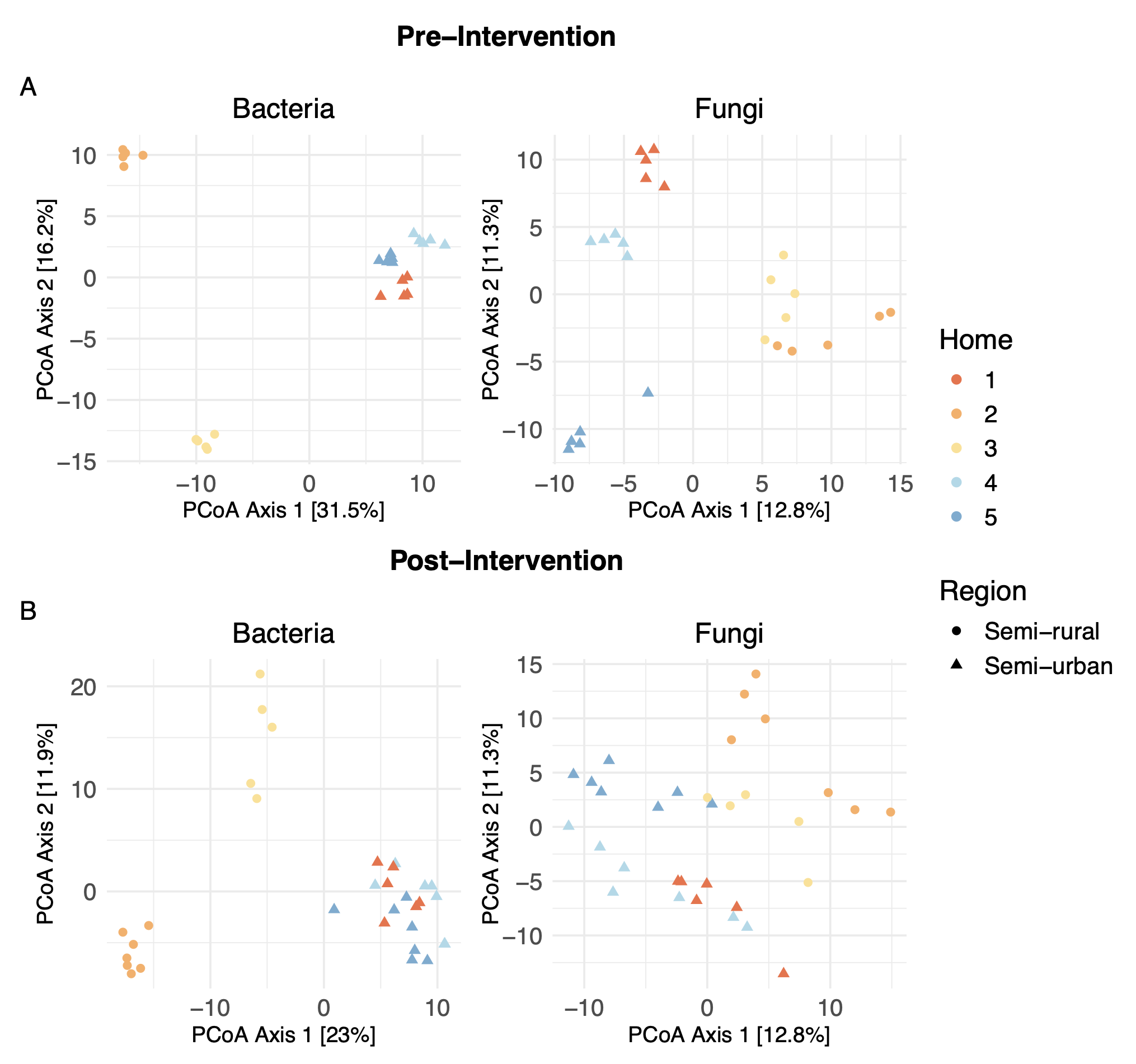


**Supplemental Figure 1. Community composition for pre- and post-intervention time periods for living room (LR) floor dust, by home and based on region of location.** Intervention homes were distinct from one another pre- and post-intervention, with differentiation between region (p < 0.001 for all comparisons; Aitchison distance; PERMANOVA).


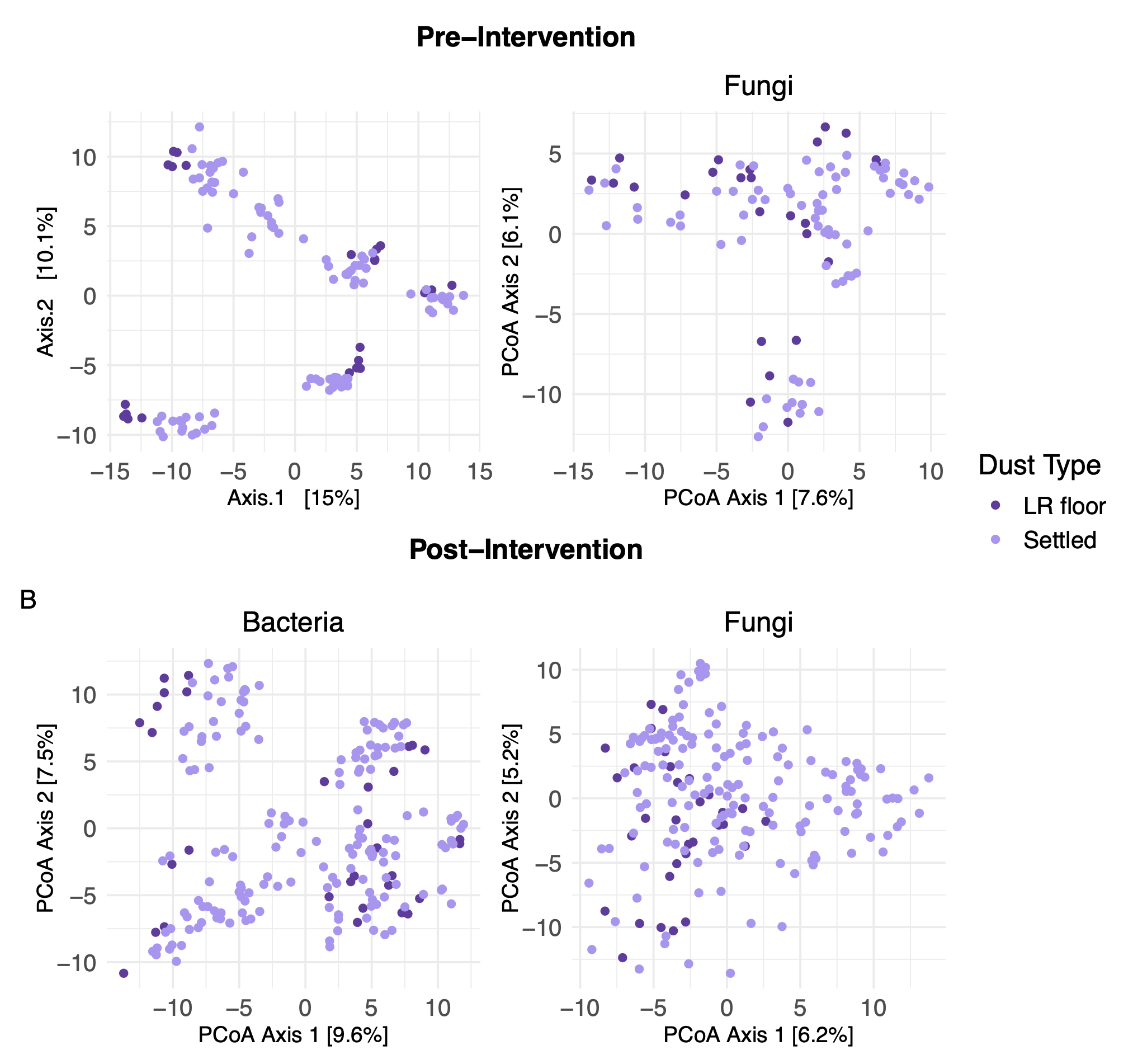


**Supplemental Figure 2. Compositional differences between living room (LR) floor dust and settled dust.** Bacteria and fungi are both significantly different between sample types for indoor samples (p < 0.001 for all comparisons; Aitchison distance; PERMANOVA). A. Pre-intervention period. B. Post-intervention period.


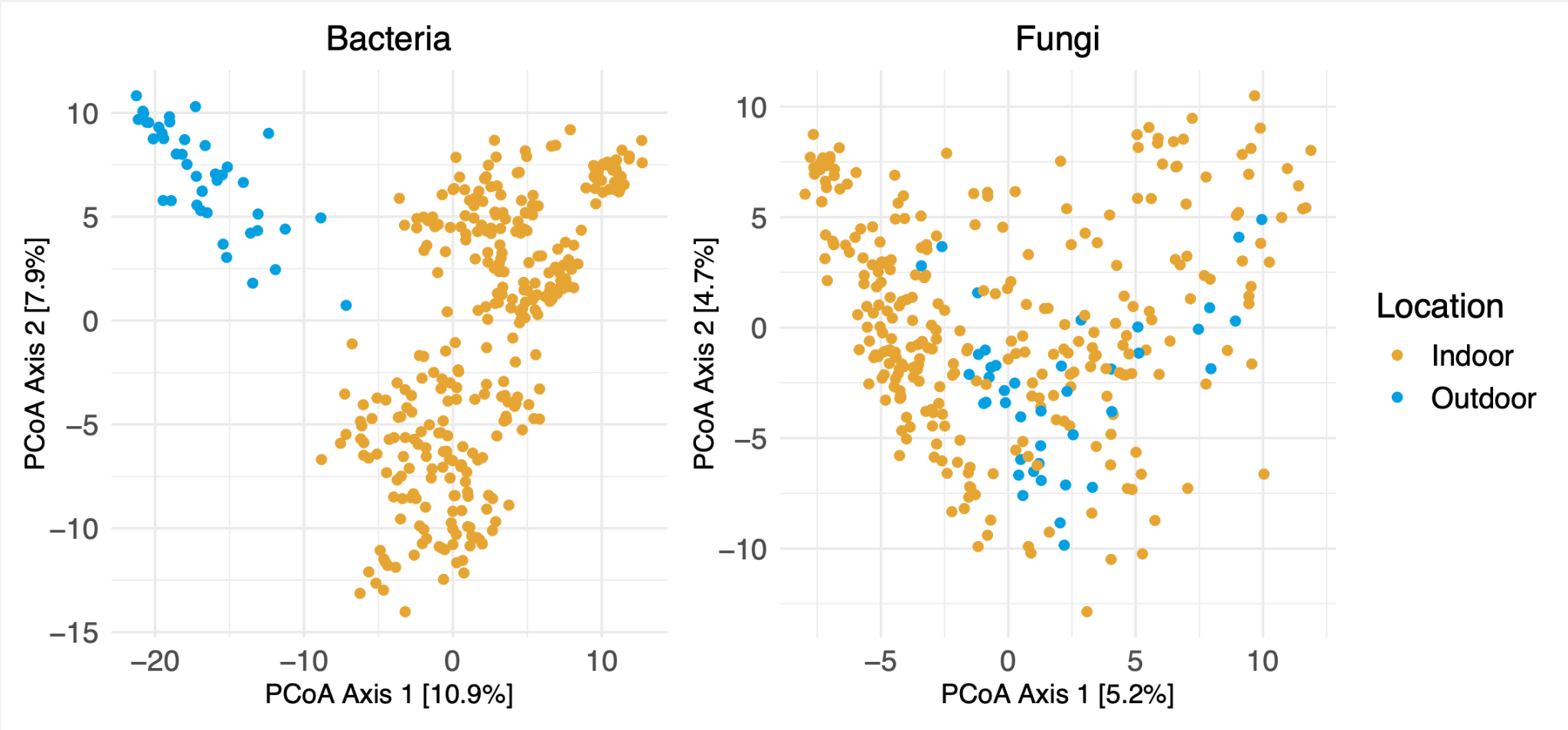


**Supplemental Figure 3. Compositional differences between outdoor and indoor samples.** Outdoor and indoor samples are unique to one another for bacterial (p < 0.001) and fungal (p < 0.001) community membership. Indoor samples include both settled and LR floor dust samples.


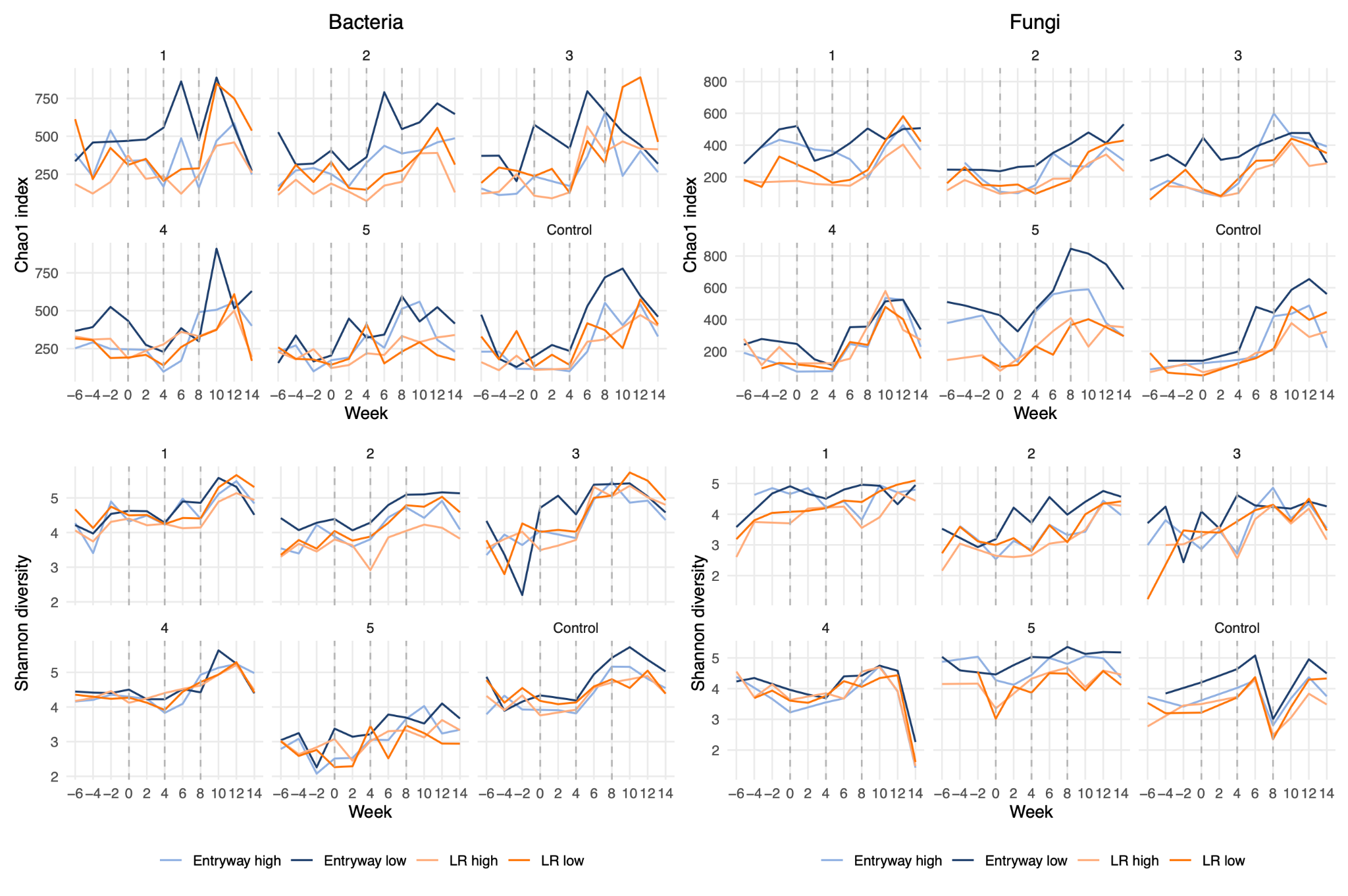


**Supplemental Figure 4. Chao1 and Shannon diversity for settled dust samples by home over time.** Dotted lines indicate intervention timepoints, for which samples were collected prior to the rug deployment. Entryway low samples were closest in proximity to the intervention rug.


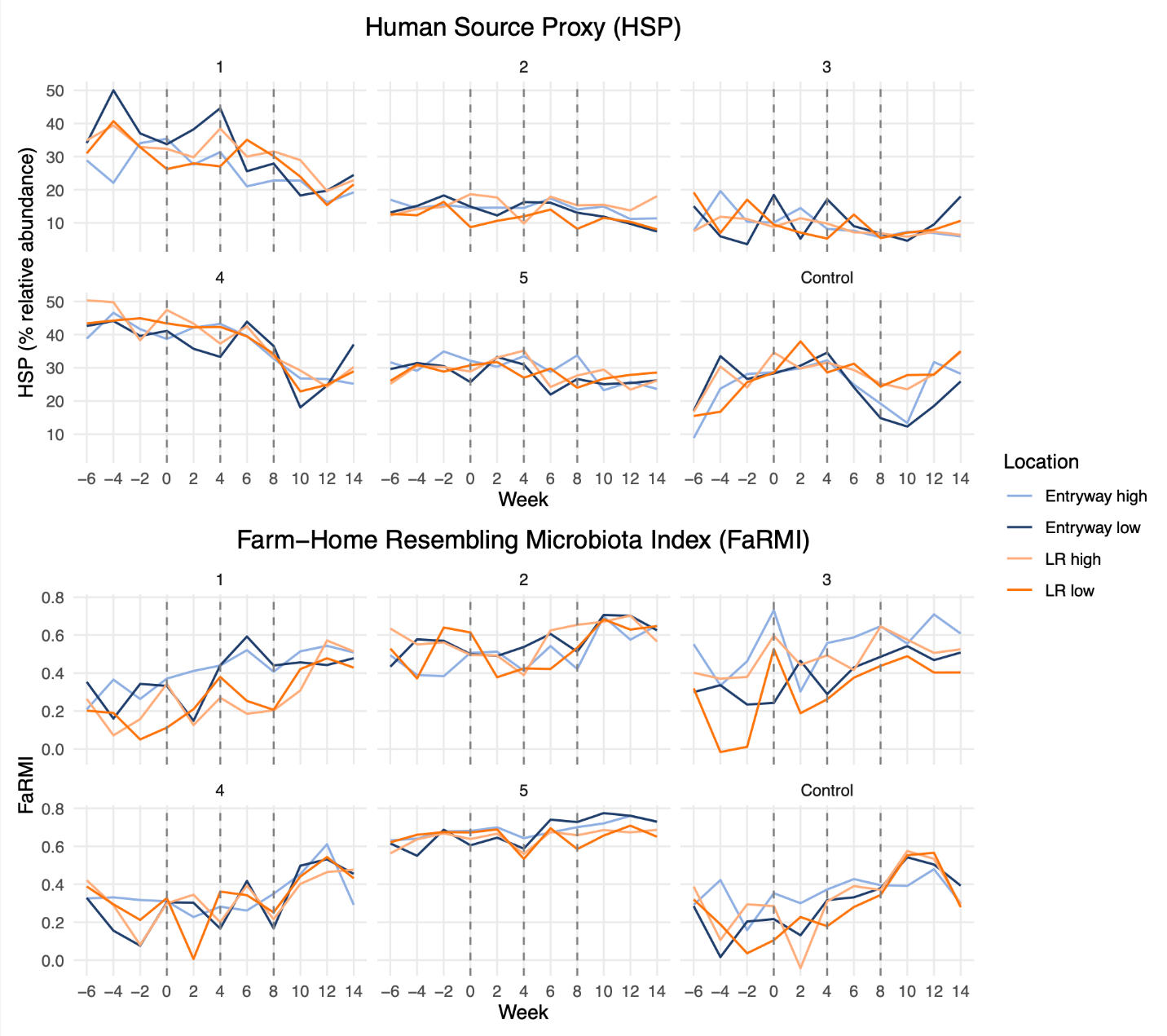


**Supplemental Figure 5. HSP and FaRMI for settled dust samples by home over time.** Dotted lines indicate intervention timepoints, for which samples were collected prior to the rug deployment. Entryway low samples were closest in proximity to the intervention rug.


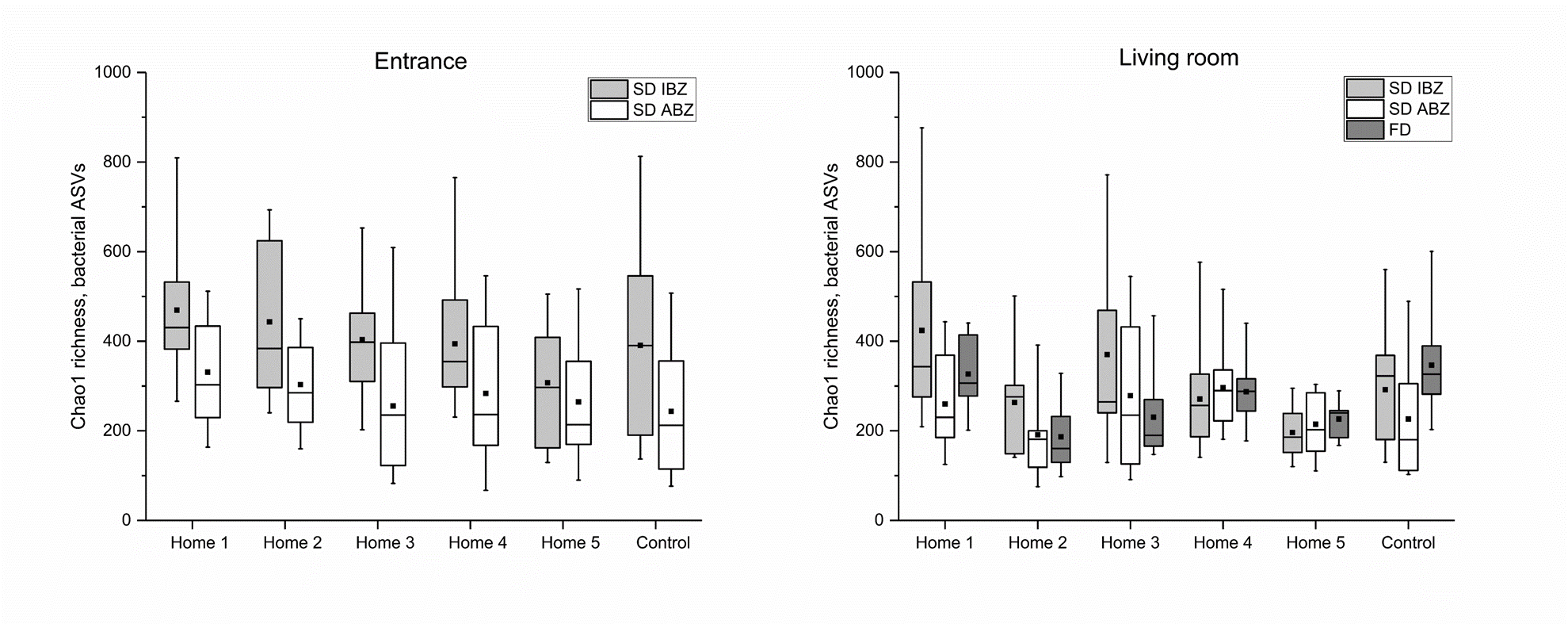
**Supplemental Figure 6. Bacterial Chao1 taxa richness by sampling location and home.** Displayed are all data points collected during baseline and intervention periods in each home and sampling location (entrance and living room settled dust infant and adult breathing zones (SD IBZ and SD ABZ, respectively), and living room floor dust (FD)). Boxes represent 25^th^, 50^th^ (median), and 75^th^ percentiles, black squares are the means, and whiskers present min and max values.


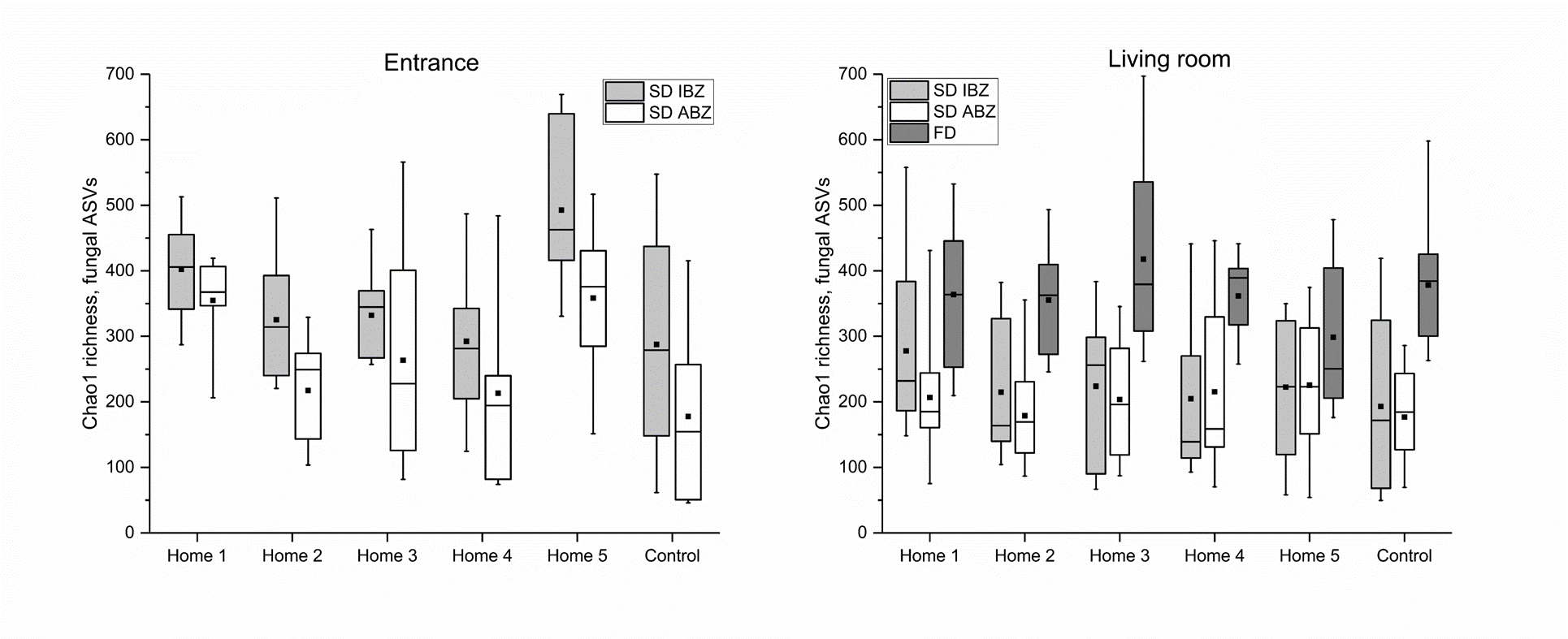


**Supplemental Figure 7. Fungal Chao1 taxa richness by sampling location and home.** Displayed are all data points collected during baseline and intervention periods in each home and sampling location (entrance and living room settled dust infant and adult breathing zones (SD IBZ and SD ABZ, respectively), and living room floor dust (FD)). Boxes represent 25^th^, 50^th^ (median), and 75^th^ percentiles, black squares are the means, and whiskers present min and max values.


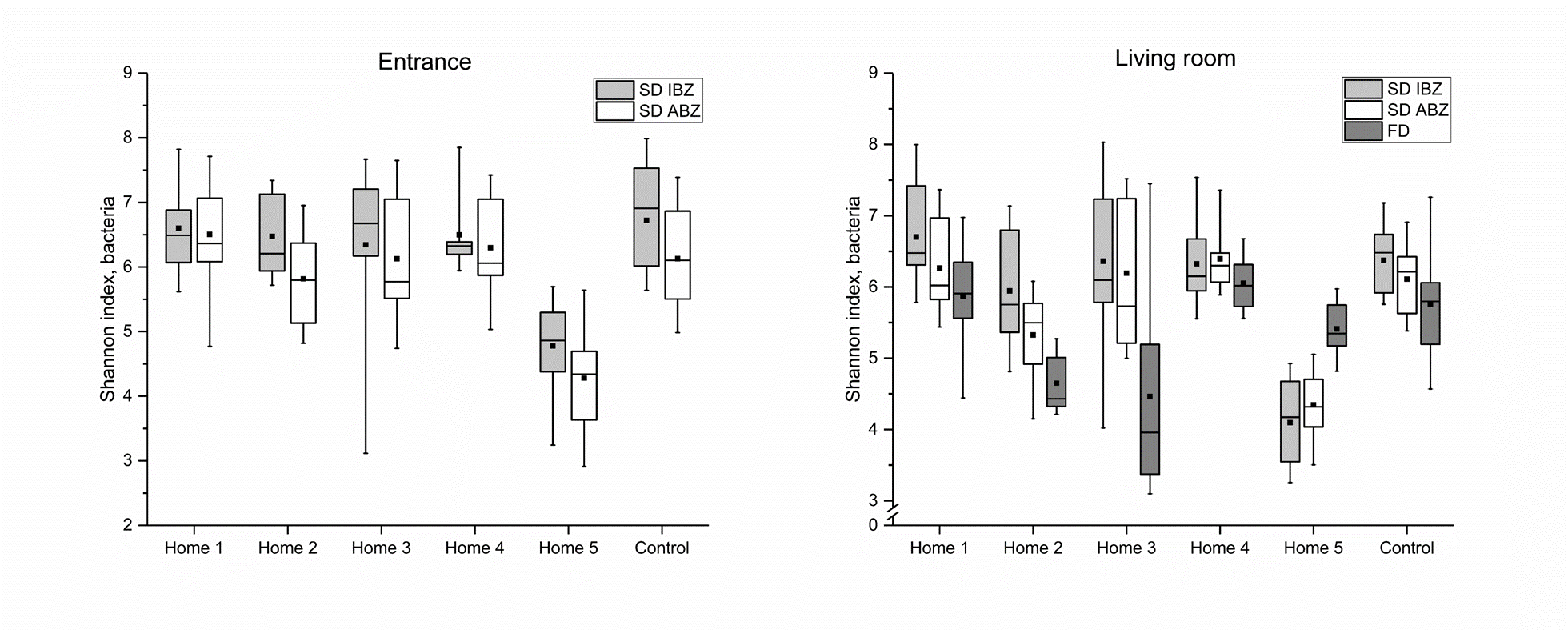


**Supplemental Figure 8. Bacterial Shannon diversity by sampling location and home.** Displayed are all data points collected during baseline and intervention periods in each home and sampling location (entrance and living room settled dust infant and adult breathing zones (SD IBZ and SD ABZ, respectively), and living room floor dust). Boxes represent 25^th^, 50^th^ (median), and 75^th^ percentiles, black squares are the means, and whiskers present min and max values.


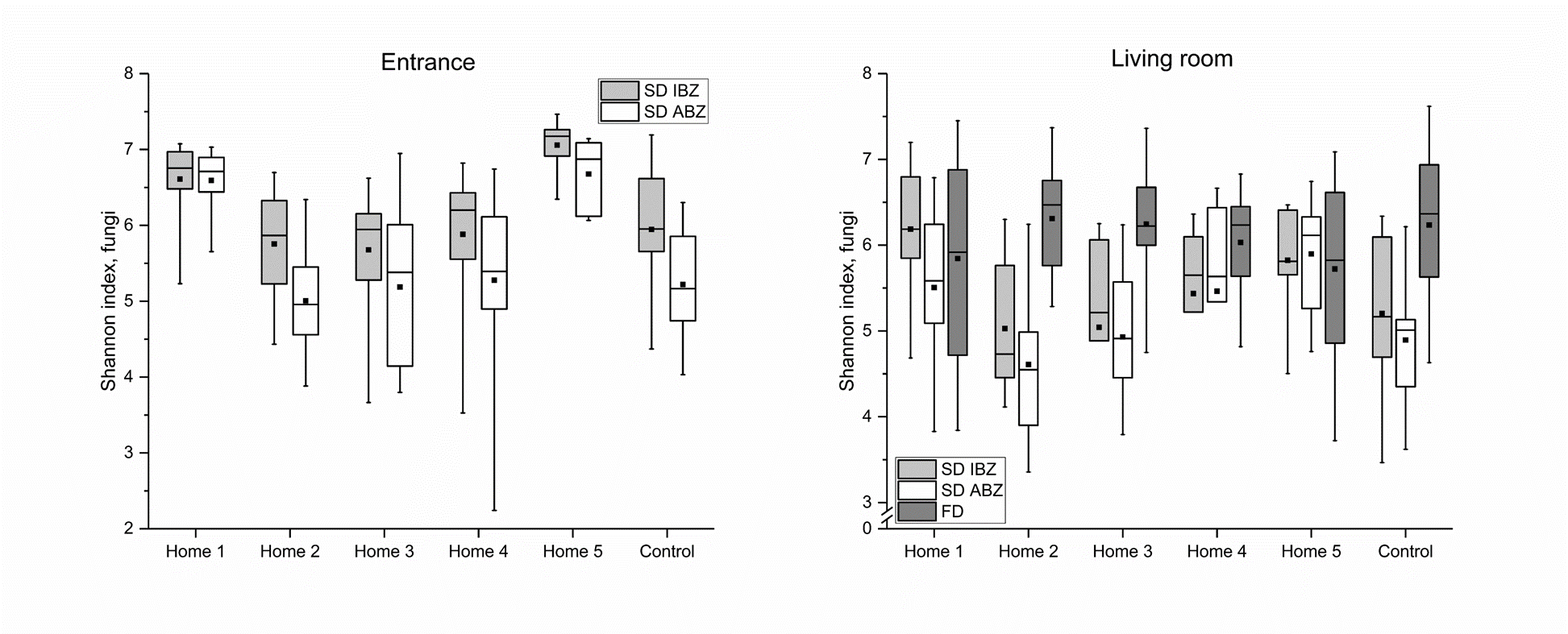


**Supplemental Figure 9. Fungal Shannon diversity by sampling location and home.** Displayed are all data points collected during baseline and intervention periods in each home and sampling location (entrance and living room settled dust infant and adult breathing zones (SD IBZ and SD ABZ, respectively), and living room floor dust). Boxes represent 25^th^, 50^th^ (median), and 75^th^ percentiles, black squares are the means, and whiskers present min and max values.


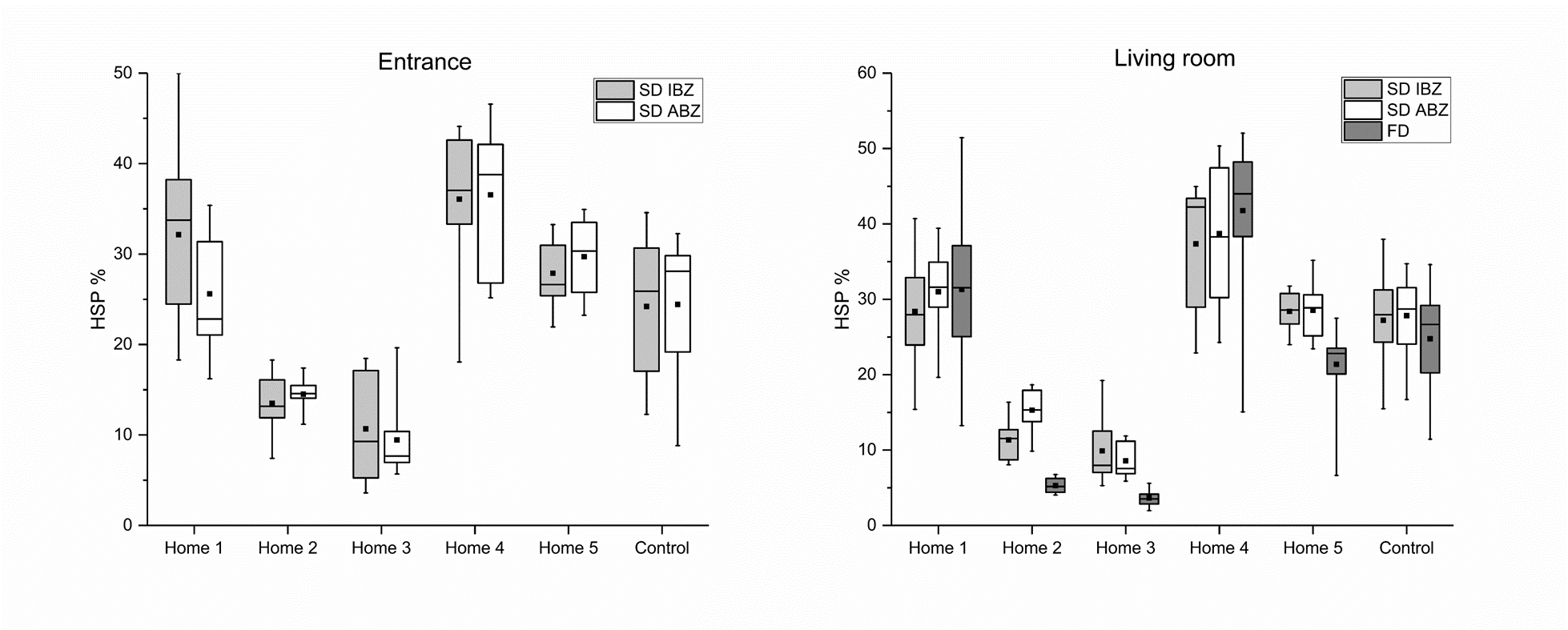


**Supplemental Figure 10. Human source proxy (HSP; %) by sampling location and home.** Displayed are all data points collected during baseline and intervention periods in each home and sampling location (entrance and living room settled dust infant and adult breathing zones (SD IBZ and SD ABZ, respectively), and living room floor dust (FD)). Boxes represent 25^th^, 50^th^ (median), and 75^th^ percentiles, black squares are the means, and whiskers present min and max values.


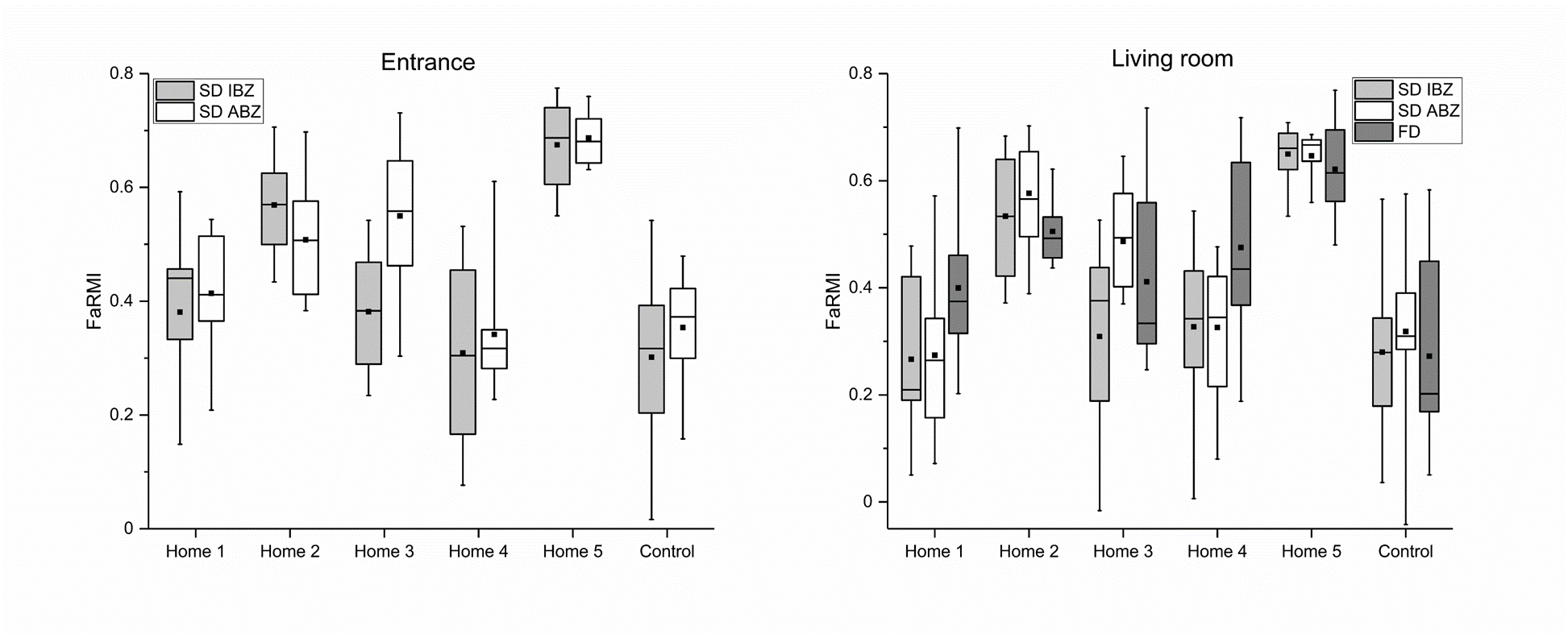


**Supplemental Figure 11. Farm home resembling microbiota index (FaRMI) by sampling location and home.** Displayed are all data points collected during baseline and intervention periods in each home and sampling location (entrance and living room settled dust infant and adult breathing zones (SD IBZ and SD ABZ, respectively), and living room floor dust (FD)). Boxes represent 25^th^, 50^th^ (median), and 75^th^ percentiles, black squares are the means, and whiskers present min and max values.

**Supplemental Tables**

**Supplemental Table 1. Bacterial and fungal taxa identified for the soil source index (SSI).** These indices were generated separately for bacteria and fungi as the sums of the relative abundances of genus level taxa that were detected across all seeding soil samples. These included 19 bacterial SSI taxa, and 28 fungal SSI taxa.

| **Bacteria SSI** |  |  |  |  |
| --- | --- | --- | --- | --- |
| **Phylum** | **Class** | **Order** | **Family** | **Genus** |
| Acidobacteriota | Acidobacteriae | **Acidobacteriales** | - | - |
| Acidobacteriota | Acidobacteriae | Solibacterales | Solibacteraceae | ***Candidatus Solibacter*** |
| Acidobacteriota | Acidobacteriae | **Subgroup_2** | - | - |
| Actinobacteriota | Acidimicrobiia | **IMCC26256** | - | - |
| Actinobacteriota | Actinobacteria | Corynebacteriales | Mycobacteriaceae | ***Mycobacterium*** |
| Actinobacteriota | Actinobacteria | Frankiales | Acidothermaceae | ***Acidothermus*** |
| Bacteroidota | Bacteroidia | Chitinophagales | Chitinophagaceae | ***Puia*** |
| Chloroflexi | Ktedonobacteria | Ktedonobacterales | **JG30-KF-AS9** | - |
| Planctomycetota | Planctomycetes | Gemmatales | **Gemmataceae** | - |
| Planctomycetota | Planctomycetes | Isosphaerales | Isosphaeraceae | ***Aquisphaera*** |
| Proteobacteria | Alphaproteobacteria | Acetobacterales | **Acetobacteraceae** | - |
| Proteobacteria | Alphaproteobacteria | **Elsterales** | - | - |
| Proteobacteria | Alphaproteobacteria | Rhizobiales | Beijerinckiaceae | ***Methylocapsa*** |
| Proteobacteria | Alphaproteobacteria | Rhizobiales | Beijerinckiaceae | ***Roseiarcus*** |
| Proteobacteria | Alphaproteobacteria | Rhizobiales | **Xanthobacteraceae** | - |
| Proteobacteria | Alphaproteobacteria | Rhizobiales | Xanthobacteraceae | ***Bradyrhizobium*** |
| Proteobacteria | Gammaproteobacteria | **WD260** | - | - |
| **RCP2-54** | - | - | - | - |
| Verrucomicrobiota | Verrucomicrobiae | Chthoniobacterales | Xiphinematobacteraceae | ***Candidatus Xiphinematobacter*** |
|  |  |  |  |  |
| **Fungi SSI** |  |  |  |  |
| **Phylum** | **Class** | **Order** | **Family** | **Genus** |
| **Ascomycota** | - | - | - | - |
| Ascomycota | **Leotiomycetes** | - | - | - |
| Ascomycota | Leotiomycetes | **Helotiales** | - | - |
| Ascomycota | Leotiomycetes | Helotiales | **Hyaloscyphaceae** | - |
| Ascomycota | Leotiomycetes | Helotiales | Hyaloscyphaceae | ***Hyaloscypha*** |
| Ascomycota | Leotiomycetes | Helotiales | Leotiaceae | ***Neobulgaria*** |
| Ascomycota | Leotiomycetes | Helotiales | Myxotrichaceae | ***Oidiodendron*** |
| Ascomycota | Leotiomycetes | Thelebolales | Pseudeurotiaceae | ***Geomyces*** |
| Ascomycota | Leotiomycetes | Thelebolales | Pseudeurotiaceae | ***Pseudeurotium*** |
| Ascomycota | Leotiomycetes | Thelebolales | Pseudeurotiaceae | ***Pseudogymnoascus*** |
| Ascomycota | Sordariomycetes | Coniochaetales | Coniochaetaceae | ***Coniochaeta*** |
| Ascomycota | Sordariomycetes | Hypocreales | Hypocreaceae | ***Trichoderma*** |
| Ascomycota | Sordariomycetes | Hypocreales | Nectriaceae | ***Neonectria*** |
| **Basidiomycota** | - | - | - | - |
| Basidiomycota | Agaricomycetes | Agaricales | Strophariaceae | ***Hypholoma*** |
| Basidiomycota | Agaricomycetes | Atheliales | Atheliaceae | ***Amphinema*** |
| Basidiomycota | Agaricomycetes | Atheliales | Atheliaceae | ***Piloderma*** |
| Basidiomycota | Agaricomycetes | Atheliales | Atheliaceae | ***Tylospora*** |
| Basidiomycota | Agaricomycetes | Russulales | Russulaceae | ***Russula*** |
| Basidiomycota | Agaricomycetes | Sebaci-les | Serendipitaceae | ***Serendipita*** |
| Basidiomycota | Agaricomycetes | Thelephorales | **Thelephoraceae** | - |
| Basidiomycota | Agaricomycetes | Thelephorales | Thelephoraceae | ***Thelephora*** |
| Basidiomycota | Microbotryomycetes | **Leucosporidiales** | - | - |
| Basidiomycota | Tremellomycetes | Filobasidiales | Piskurozymaceae | ***Solicoccozyma*** |
| Mortierellomycota | Mortierellomycetes | Mortierellales | Mortierellaceae | ***Mortierella*** |
| Mucoromycota | Umbelopsidomycetes | Umbelopsidales | Umbelopsidaceae | ***Umbelopsis*** |
| Rozellomycota | Rozellomycoti-_cls_Incertae_sedis | **GS11** | - | - |
| **Unassigned** |  |  |  |  |

**Supplemental Table 2. Farm-home resembling microbiota index (FaRMI)-associated taxa.** The FaRMI is indicative of the asthma-protective potential of the microbial community and was calculated as the individual probability score of a sample being from a farm or rural non-farm home, based on logistic regression analysis of four main PCoA axes from generalized UniFrac analysis.

| **Phylum** | **Class** | **Order** | **Family** | **Genus** | **Coefficient** |
| --- | --- | --- | --- | --- | --- |
| **Intercept** | **-** | **-** | **-** | **-** | 1.10E-01 |
| Acidobacteria | Acidobacteria-6 | iii1-15 | **mb2424** | - | -1.10E-03 |
| **Actinobacteria** | - | - | - | - | 3.40E-03 |
| Actinobacteria | Actinobacteria | Actinomycetales | Micromonosporaceae | ***Dactylosporangium*** | 9.00E-04 |
| Actinobacteria | Actinobacteria | Bifidobacteriales | Bifidobacteriaceae | ***Gardnerella*** | -4.40E-04 |
| **Bacteroidetes** | - | - | - | - | 1.70E-03 |
| Bacteroidetes | Bacteroidia | Bacteroidales | **Bacteroidaceae** | - | 2.00E-04 |
| Bacteroidetes | Flavobacteriia | Flavobacteriales | [Weeksellaceae] | ***Chryseobacterium*** | 3.80E-04 |
| Bacteroidetes | Flavobacteriia | Flavobacteriales | [Weeksellaceae] | ***Ornithobacterium*** | 9.30E-04 |
| Bacteroidetes | **Sphingobacteriia** | - | - | - | 5.40E-06 |
| **Cyanobacteria** | - | - | - | - | 8.90E-04 |
| Cyanobacteria | Chloroplast | **Chlorophyta** | - | - | 6.90E-04 |
| Firmicutes | **Bacilli** | - | - | - | -1.70E-03 |
| Firmicutes | Bacilli | Bacillales | **Listeriaceae** | - | -7.40E-04 |
| Firmicutes | Bacilli | Bacillales | **Planococcaceae** | - | 6.60E-05 |
| Firmicutes | Bacilli | Bacillales | Staphylococcaceae | ***Staphylococcus*** | 4.00E-04 |
| Firmicutes | Bacilli | Lactobacillales | **Streptococcaceae** | - | -2.90E-03 |
| Fusobacteria | Fusobacteriia | Fusobacteriales | **Fusobacteriaceae** | - | -4.90E-04 |
| Proteobacteria | [Saprospirae] | [Saprospirales] | Chitinophagaceae | ***Flavihumibacter*** | 1.00E-03 |
| Proteobacteria | Actinobacteria | Actinomycetales | Dermabacteraceae | ***Brachybacterium*** | 3.20E-04 |
| Proteobacteria | Actinobacteria | Actinomycetales | **Micrococcaceae** | - | 3.60E-04 |
| Proteobacteria | Actinobacteria | Actinomycetales | **Yaniellaceae** | - | 4.70E-04 |
| Proteobacteria | **Alphaproteobacteria** | - | - | - | 8.30E-04 |
| Proteobacteria | Alphaproteobacteria | **Caulobacterales** | - | - | 6.60E-04 |
| Proteobacteria | Alphaproteobacteria | Caulobacterales | Caulobacteraceae | ***Brevundimonas*** | -4.00E-04 |
| Proteobacteria | Alphaproteobacteria | Sphingomonadales | **Sphingomonadaceae** | - | 1.10E-03 |
| Proteobacteria | Alphaproteobacteria | unassigned | unassigned | **unassigned** | -7.20E-04 |
| Proteobacteria | Bacilli | Bacillales | **Staphylococcaceae** | - | 2.00E-04 |
| Proteobacteria | Bacilli | Lactobacillales | Streptococcaceae | ***Streptococcus*** | 3.40E-04 |
| Proteobacteria | Bacteroidia | Bacteroidales | [Odoribacteraceae] | ***Butyricimonas*** | 7.10E-04 |
| Proteobacteria | Bacteroidia | Bacteroidales | **Porphyromonadaceae** | - | -3.80E-04 |
| Proteobacteria | **Betaproteobacteria** | - | - | - | -5.40E-04 |
| Proteobacteria | Betaproteobacteria | Burkholderiales | Alcaligenaceae | ***Alcaligenes*** | 5.90E-04 |
| Proteobacteria | Betaproteobacteria | Burkholderiales | Comamonadaceae | ***Comamonas*** | 2.30E-04 |
| Proteobacteria | Betaproteobacteria | Burkholderiales | **Oxalobacteraceae** | - | 3.80E-04 |
| Proteobacteria | Betaproteobacteria | **Methylophilales** | - | - | 4.60E-04 |
| Proteobacteria | Betaproteobacteria | **Rhodocyclales** | - | - | 6.10E-04 |
| Proteobacteria | Clostridia | Clostridiales | Lachnospiraceae | ***Lachnobacterium*** | -4.10E-04 |
| Proteobacteria | Coriobacteriia | Coriobacteriales | Coriobacteriaceae | ***Eggerthella*** | -5.60E-04 |
| Proteobacteria | Deinococci | Deinococcales | **Trueperaceae** | - | -2.20E-04 |
| Proteobacteria | Deltaproteobacteria | Myxococcales | Myxococcaceae | ***Corallococcus*** | 1.10E-03 |
| Proteobacteria | Flavobacteriia | Flavobacteriales | Flavobacteriaceae | ***Winogradskyella*** | 6.50E-04 |
| Proteobacteria | Gammaproteobacteria | Enterobacteriales | Enterobacteriaceae | ***Dickeya*** | -1.10E-03 |
| Proteobacteria | **Gemm-3** | - | - | - | -6.60E-04 |
| Proteobacteria | Solibacteres | Solibacterales | **Solibacteraceae** | - | 4.30E-04 |
| **Unassigned** |  |  |  |  | -8.00E-04 |

**Supplemental Table 3. Statistical results for multivariate analysis.** Data show the relative importance of home characteristics and occupant demographics on microbial communities, separately for pre- and post-intervention samples, location of sample collection, and taxonomic group. Results were generated using a PERMANOVA, using marginal testing to ensure variable independence. R^2^ represents the proportion of variance explained by each predictor variable, and F-values represent the ratio of explained variance to unexplained variance.

| **Intervention Period** | **Sample Type** | **Taxon** | **Variable** | **P-value** | **R^2^** | **F-value** |
| --- | --- | --- | --- | --- | --- | --- |
| Pre-Intervention | Settled Dust | Bacteria | Ventilation | < 0.001 | 0.14953 | 11.1462 |
| Pre-Intervention | Settled Dust | Bacteria | Occupants | < 0.001 | 0.09167 | 13.666 |
| Pre-Intervention | Settled Dust | Bacteria | Dogs | < 0.001 | 0.07258 | 10.82 |
| Pre-Intervention | Settled Dust | Bacteria | Region | < 0.001 | 0.06481 | 9.6619 |
| Pre-Intervention | Settled Dust | Fungi | Ventilation | < 0.001 | 0.04746 | 4.143 |
| Pre-Intervention | Settled Dust | Fungi | Occupants | < 0.001 | 0.08076 | 3.5248 |
| Pre-Intervention | Settled Dust | Fungi | Dogs | < 0.001 | 0.03893 | 3.3986 |
| Pre-Intervention | Settled Dust | Fungi | Region | 0.002 | 0.05658 | 4.9387 |
| Pre-Intervention | LR Floor Dust | Bacteria | Ventilation | < 0.001 | 0.27524 | 8.9615 |
| Pre-Intervention | LR Floor Dust | Bacteria | Occupants | < 0.001 | 0.14515 | 9.4518 |
| Pre-Intervention | LR Floor Dust | Bacteria | Dogs | < 0.001 | 0.24794 | 16.1453 |
| Pre-Intervention | LR Floor Dust | Bacteria | Region | < 0.001 | 0.24794 | 16.1453 |
| Pre-Intervention | LR Floor Dust | Fungi | Ventilation | < 0.001 | 0.18358 | 3.0938 |
| Pre-Intervention | LR Floor Dust | Fungi | Occupants | < 0.001 | 0.09967 | 3.3594 |
| Pre-Intervention | LR Floor Dust | Fungi | Dogs | < 0.001 | 0.11216 | 3.7803 |
| Pre-Intervention | LR Floor Dust | Fungi | Region | < 0.001 | 0.11216 | 3.7803 |
| Post-Intervention | Settled Dust | Bacteria | Ventilation | < 0.001 | 0.09513 | 10.324 |
| Post-Intervention | Settled Dust | Bacteria | Occupants | < 0.001 | 0.0633 | 13.74 |
| Post-Intervention | Settled Dust | Bacteria | Dogs | < 0.001 | 0.05477 | 11.888 |
| Post-Intervention | Settled Dust | Bacteria | Region | < 0.001 | 0.04976 | 10.8 |
| Post-Intervention | Settled Dust | Fungi | Ventilation | < 0.001 | 0.0588 | 5.2874 |
| Post-Intervention | Settled Dust | Fungi | Occupants | < 0.001 | 0.0316 | 5.684 |
| Post-Intervention | Settled Dust | Fungi | Dogs | < 0.001 | 0.03162 | 5.6875 |
| Post-Intervention | Settled Dust | Fungi | Region | < 0.001 | 0.02221 | 3.9949 |
| Post-Intervention | LR Floor Dust | Bacteria | Ventilation | < 0.001 | 0.19642 | 5.0429 |
| Post-Intervention | LR Floor Dust | Bacteria | Occupants | < 0.001 | 0.10235 | 5.2553 |
| Post-Intervention | LR Floor Dust | Bacteria | Dogs | < 0.001 | 0.16339 | 8.3895 |
| Post-Intervention | LR Floor Dust | Bacteria | Region | < 0.001 | 0.16339 | 8.3895 |
| Post-Intervention | LR Floor Dust | Fungi | Ventilation | < 0.001 | 0.12259 | 2.2598 |
| Post-Intervention | LR Floor Dust | Fungi | Occupants | < 0.001 | 0.0685 | 2.5256 |
| Post-Intervention | LR Floor Dust | Fungi | Dogs | < 0.001 | 0.07045 | 2.5975 |
| Post-Intervention | LR Floor Dust | Fungi | Region | < 0.001 | 0.07045 | 2.5975 |

**Supplemental Table 4. Top 50 most abundant bacterial and fungal, genus level taxa in the seeding soil.** Taxa are sorted descending by mean relative abundance across seeding soil samples.

| **Phylum** | **Class** | **Order** | **Family** | **Genus** | **Mean RA** |
| --- | --- | --- | --- | --- | --- |
| **BACTERIA** |  |  |  |  |  |
| Proteobacteria | Alphaproteobacteria | Rhizobiales | Xanthobacteraceae | Bradyrhizobium | 9.15% |
| Acidobacteriota | Acidobacteriae | Subgroup_2 | NA | NA | 7.53% |
| Proteobacteria | Alphaproteobacteria | Rhizobiales | Xanthobacteraceae | NA | 7.45% |
| Actinobacteriota | Actinobacteria | Frankiales | Acidothermaceae | Acidothermus | 6.11% |
| Proteobacteria | Gammaproteobacteria | WD260 | NA | NA | 5.50% |
| Proteobacteria | Alphaproteobacteria | Elsterales | NA | NA | 5.30% |
| Proteobacteria | Alphaproteobacteria | Rhizobiales | Beijerinckiaceae | Roseiarcus | 4.48% |
| Acidobacteriota | Acidobacteriae | Acidobacteriales | NA | NA | 4.45% |
| Proteobacteria | Alphaproteobacteria | Acetobacterales | Acetobacteraceae | NA | 3.30% |
| Actinobacteriota | Actinobacteria | Corynebacteriales | Mycobacteriaceae | Mycobacterium | 2.58% |
| Acidobacteriota | Acidobacteriae | Solibacterales | Solibacteraceae | Candidatus_Solibacter | 2.27% |
| Planctomycetota | Planctomycetes | Gemmatales | Gemmataceae | NA | 2.21% |
| Bacteroidota | Bacteroidia | Chitinophagales | Chitinophagaceae | Puia | 2.03% |
| RCP2-54 | NA | NA | NA | NA | 2.01% |
| Acidobacteriota | Acidobacteriae | Bryobacterales | Bryobacteraceae | Bryobacter | 1.89% |
| Verrucomicrobiota | Verrucomicrobiae | Chthoniobacterales | Xiphinematobacteraceae | Cand._Xiphinematobacter | 1.46% |
| Proteobacteria | Alphaproteobacteria | Micropepsales | Micropepsaceae | NA | 1.28% |
| Proteobacteria | Alphaproteobacteria | Rhizobiales | Beijerinckiaceae | Methylocapsa | 1.28% |
| Acidobacteriota | Vicinamibacteria | Vicinamibacterales | NA | NA | 1.11% |
| Planctomycetota | Planctomycetes | Isosphaerales | Isosphaeraceae | Aquisphaera | 1.03% |
| Proteobacteria | Gammaproteobacteria | Gammaproteobacteria_Incertae_Sedis | Unknown_Family | Acidibacter | 0.97% |
| Acidobacteriota | Acidobacteriae | Acidobacteriales | Acidobacteriaceae_(Subgroup_1) | Granulicella | 0.94% |
| Actinobacteriota | Thermoleophilia | Solirubrobacterales | Solirubrobacteraceae | NA | 0.91% |
| Myxococcota | Polyangia | Polyangiales | Polyangiaceae | Pajaroellobacter | 0.79% |
| Verrucomicrobiota | Verrucomicrobiae | Chthoniobacterales | Chthoniobacteraceae | Candidatus_Udaeobacter | 0.78% |
| WPS-2 | NA | NA | NA | NA | 0.73% |
| Actinobacteriota | Acidimicrobiia | IMCC26256 | NA | NA | 0.71% |
| Proteobacteria | Alphaproteobacteria | NA | NA | NA | 0.69% |
| Planctomycetota | Planctomycetes | Isosphaerales | Isosphaeraceae | NA | 0.67% |
| Chloroflexi | Ktedonobacteria | Ktedonobacterales | JG30-KF-AS9 | NA | 0.61% |
| Acidobacteriota | Acidobacteriae | Acidobacteriales | Koribacteraceae | Candidatus_Koribacter | 0.57% |
| Firmicutes | Clostridia | Clostridiales | Clostridiaceae | Clostridium_sensu_stricto_13 | 0.54% |
| Chloroflexi | AD3 | NA | NA | NA | 0.53% |
| Actinobacteriota | Acidimicrobiia | NA | NA | NA | 0.52% |
| Bacteroidota | Bacteroidia | Sphingobacteriales | Sphingobacteriaceae | Mucilaginibacter | 0.50% |
| Proteobacteria | Gammaproteobacteria | JG36-TzT-191 | NA | NA | 0.50% |
| Planctomycetota | Planctomycetes | Pirellulales | Pirellulaceae | NA | 0.49% |
| Acidobacteriota | Acidobacteriae | Acidobacteriales | Acidobacteriaceae_(Subgroup_1) | Acidipila | 0.48% |
| Actinobacteriota | Thermoleophilia | Gaiellales | NA | NA | 0.48% |
| Myxococcota | Polyangia | Haliangiales | Haliangiaceae | Haliangium | 0.48% |
| Verrucomicrobiota | Verrucomicrobiae | Pedosphaerales | Pedosphaeraceae | NA | 0.46% |
| NA | NA | NA | NA | NA | 0.42% |
| Proteobacteria | Alphaproteobacteria | Rhizobiales | Xanthobacteraceae | Rhodoplanes | 0.40% |
| Proteobacteria | Alphaproteobacteria | Caulobacterales | Caulobacteraceae | NA | 0.39% |
| Gemmatimonadota | Gemmatimonadetes | Gemmatimonadales | Gemmatimonadaceae | NA | 0.38% |
| Planctomycetota | Phycisphaerae | Tepidisphaerales | WD2101_soil_group | NA | 0.36% |
| Actinobacteriota | Thermoleophilia | Solirubrobacterales | Solirubrobacteraceae | Conexibacter | 0.36% |
| Proteobacteria | Gammaproteobacteria | Burkholderiales | Burkholderiaceae | Burkholderia-Caballeronia-Paraburkholderia | 0.34% |
| Acidobacteriota | Holophagae | Subgroup_7 | NA | NA | 0.30% |
| Firmicutes | Clostridia | Clostridiales | Clostridiaceae | Clostridium_sensu_stricto_9 | 0.30% |
|  |  |  |  |  |  |
| **FUNGI** |  |  |  |  |  |
| Basidiomycota | Agaricomycetes | Atheliales | Atheliaceae | Piloderma | 25.29% |
| Mortierellomycota | Mortierellomycetes | Mortierellales | Mortierellaceae | Mortierella | 12.81% |
| Basidiomycota | Agaricomycetes | Russulales | Russulaceae | Russula | 6.62% |
| Basidiomycota | Tremellomycetes | Filobasidiales | Piskurozymaceae | Solicoccozyma | 6.05% |
| Basidiomycota | Agaricomycetes | Atheliales | Atheliaceae | Tylospora | 5.30% |
| Ascomycota | Sordariomycetes | Coniochaetales | Coniochaetaceae | Coniochaeta | 4.84% |
| Rozellomycota | Rozellomycotina_cls_Incertae_sedis | GS11 | NA | NA | 3.65% |
| Basidiomycota | Agaricomycetes | Sebacinales | Serendipitaceae | Serendipita | 2.96% |
| Ascomycota | Leotiomycetes | Thelebolales | Pseudeurotiaceae | Pseudeurotium | 2.38% |
| Ascomycota | NA | NA | NA | NA | 2.37% |
| Ascomycota | Leotiomycetes | NA | NA | NA | 2.32% |
| NA | NA | NA | NA | NA | 2.16% |
| Basidiomycota | Agaricomycetes | Thelephorales | Thelephoraceae | NA | 2.02% |
| Ascomycota | Leotiomycetes | Helotiales | NA | NA | 1.87% |
| Basidiomycota | Microbotryomycetes | Leucosporidiales | NA | NA | 1.32% |
| Basidiomycota | NA | NA | NA | NA | 1.19% |
| Ascomycota | Leotiomycetes | Helotiales | Myxotrichaceae | Oidiodendron | 1.15% |
| Ascomycota | Archaeorhizomycetes | Archaeorhizomycetales | Archaeorhizomycetaceae | Archaeorhizomyces | 1.07% |
| Ascomycota | Saccharomycetes | NA | NA | NA | 1.06% |
| Basidiomycota | Agaricomycetes | Thelephorales | Thelephoraceae | Thelephora | 0.77% |
| Ascomycota | Eurotiomycetes | Eurotiales | Aspergillaceae | Penicillium | 0.77% |
| Basidiomycota | Agaricomycetes | Agaricales | Strophariaceae | Hypholoma | 0.74% |
| Mucoromycota | Umbelopsidomycetes | Umbelopsidales | Umbelopsidaceae | Umbelopsis | 0.73% |
| Basidiomycota | Agaricomycetes | Atheliales | Atheliaceae | Amphinema | 0.72% |
| Ascomycota | Leotiomycetes | Helotiales | Leotiaceae | Neobulgaria | 0.51% |
| Ascomycota | Sordariomycetes | Hypocreales | Nectriaceae | Neonectria | 0.49% |
| Basidiomycota | Agaricomycetes | Agaricales | Clavariaceae | Ramariopsis | 0.42% |
| Ascomycota | Leotiomycetes | Thelebolales | Pseudeurotiaceae | Geomyces | 0.42% |
| Ascomycota | Leotiomycetes | Helotiales | Hyaloscyphaceae | NA | 0.40% |
| Ascomycota | Dothideomycetes | Pleosporales | Melanommataceae | Pleotrichocladium | 0.38% |
| Ascomycota | Leotiomycetes | Helotiales | Helotiales_fam_Incertae_sedis | Leptodontidium | 0.38% |
| Ascomycota | Leotiomycetes | Helotiales | Hyaloscyphaceae | Hyaloscypha | 0.37% |
| Ascomycota | Leotiomycetes | Helotiales | Helotiales_fam_Incertae_sedis | Xenopolyscytalum | 0.35% |
| Ascomycota | Leotiomycetes | Thelebolales | Pseudeurotiaceae | Pseudogymnoascus | 0.26% |
| Basidiomycota | Agaricomycetes | Agaricales | Cortinariaceae | Cortinarius | 0.23% |
| Ascomycota | Sordariomycetes | Hypocreales | Hypocreaceae | Trichoderma | 0.22% |
| Basidiomycota | Agaricomycetes | Thelephorales | Thelephoraceae | Tomentella | 0.21% |
| Basidiomycota | Microbotryomycetes | Sporidiobolales | Sporidiobolaceae | Rhodosporidiobolus | 0.21% |
| Ascomycota | Leotiomycetes | Helotiales | Hyaloscyphaceae | Lachnellula | 0.20% |
| Ascomycota | Leotiomycetes | Helotiales | Hyaloscyphaceae | Hyphodiscus | 0.20% |
| Basidiomycota | Agaricomycetes | Agaricales | Hydnangiaceae | Laccaria | 0.19% |
| Ascomycota | Pezizomycetes | Pezizales | Pyronemataceae | Scutellinia | 0.15% |
| Ascomycota | Pezizomycetes | Pezizales | Pyronemataceae | Byssonectria | 0.15% |
| Ascomycota | Pezizomycetes | Pezizales | Tuberaceae | Tuber | 0.14% |
| Ascomycota | Sordariomycetes | Chaetosphaeriales | Chaetosphaeriaceae | NA | 0.14% |
| Ascomycota | Leotiomycetes | Helotiales | Hyaloscyphaceae | Hyalopeziza | 0.12% |
| Basidiomycota | Agaricomycetes | NA | NA | NA | 0.12% |
| Basidiomycota | Tremellomycetes | Tremellales | Trimorphomycetaceae | Saitozyma | 0.12% |
| Ascomycota | Leotiomycetes | Helotiales | Vibrisseaceae | Phialocephala | 0.12% |
| Basidiomycota | Tremellomycetes | Cystofilobasidiales | Mrakiaceae | Krasilnikovozyma | 0.12% |

**Supplemental Table 5. Comparison of bacterial and fungal levels determined with qPCR in paired pre- versus post-soil seeding samples.** Three sample pairs were generated from each home, utilizing the three intervention time points, comparing the two-week measurement preceding the seeding event to the two weeks after (pre2w-post2w), as well as the two weeks before seeding to the four weeks after (pre2w-post4w). P-values were calculated with Student's t-test or Wilcoxon Signed Rank test (in case of not normally distributed data) and are bolded to indicate statistical significance at p < 0.05. ‘Mean diff.’ represents the mean of the differences between paired pre/post samples, where positive values indicate increases, and negative values decreases in post-seeding intervention compared to before. Measurement values are presented with three significant figures.

|  |  | **Entrance IBZ (n=13-14)** | | **Entrance ABZ (n=15)** | | **LR IBZ (n=15)** | | **LR ABZ (n=14-15)** | | **LR floor dust (n=13-14)** | |
| --- | --- | --- | --- | --- | --- | --- | --- | --- | --- | --- | --- |
|  |  | Mean diff. | *p-value* | Mean diff. | *p-value* | Mean diff. | *p-value* | Mean diff. | *p-value* | Mean diff. | *p-value* |
| **Gram-positive** | pre2w -post2w | -1210 000 | *0.194* | 33 400 | *0.603* | 115 000 | *0.279* | -23 700 | *0.713* | -101 000 | *0.502* |
|  | pre2w -post4w | -770 000 | *0.414* | 20 500 | *0.804* | 5 670 | *0.961* | -7 260 | *0.944* | -148 000 | *0.532* |
| **Gram-negative** | pre2w -post2w | 201 000 | *0.358* | 153 000 | ***0.022*** | 439 000 | *0.064* | 147 000 | ***0.040*** | 110 000 | *0.613* |
|  | pre2w -post4w | 2 320 000 | *0.273* | 1 200 000 | *0.135* | 858 000 | *0.058* | 388 000 | *0.151* | 386 000 | *0.383* |
| **Total fungi** | pre2w -post2w | 7 640 | *0.613* | 511 | *0.639* | 13 100 | ***0.015*** | 2 250 | *0.389* | 2 750 | *0.463* |
|  | pre2w -post4w | 10 100 | *0.510* | 10 300 | *0.252* | 10 200 | ***0.015*** | 6 120 | *0.180* | 3 040 | *0.515* |

**Supplemental Table 6. Comparison of microbial measurements in paired pre- versus post-soil seeding samples in the control home.** Three sample pairs were generated according to the three intervention time points, comparing the two-week measurement preceding the seeding event to the two weeks after (pre2w-post2w), as well as the two weeks before seeding to the four weeks after (pre2w-post4w). No soil seeding was carried out in this control home, but samples were collected at same time points as in the intervention homes. ‘Mean diff.’ represents the mean of the differences between paired pre/post samples, where positive values indicate increases, and negative values decreases in post-seeding intervention compared to before. Measurement values are presented with three significant figures. (SSI, soil source index; Chao1, Chao1 richness estimate; Shannon, Shannon diversity; HSP, humans source proxy; FaRMI, farm-home resembling microbiota index; IBZ, infant breathing zone; ABZ, adult breathing zone; LR, living room.)

|  |  | **Entrance IBZ (n=3)** | **Entrance ABZ (n=3)** | **LR IBZ (n=3)** | **LR ABZ (n=3)** | **LR floor dust (n=3)** |
| --- | --- | --- | --- | --- | --- | --- |
|  |  | Mean diff. | Mean diff. | Mean diff. | Mean diff. | Mean diff. |
| **Bacteria SSI** | pre2w - post2w | 0.08 | -0.03 | -0.35 | -0.29 | 0.94 |
|  | pre2w - post4w | 0.40 | 0.40 | 0.68 | 0.37 | 0.37 |
|  |  |  |  |  |  |  |
| **Bacteria Chao** | pre2w - post2w | 165 | 3.03 | 57.2 | -20.2 | -46.8 |
|  | pre2w - post4w | 118 | 121 | 143 | 126 | -16.8 |
|  |  |  |  |  |  |  |
| **Bacteria Shannon** | pre2w - post2w | 0.41 | 0.44 | -0.02 | 0.04 | 0.38 |
|  | pre2w - post4w | 0.44 | 0.63 | 0.39 | 0.51 | 0.47 |
|  |  |  |  |  |  |  |
| **HSP** | pre2w - post2w | -3.54 | -4.00 | 5.18 | -1.98 | -1.28 |
|  | pre2w - post4w | -3.27 | 1.02 | -0.20 | -2.17 | -2.68 |
|  |  |  |  |  |  |  |
| **FaRMI** | pre2w - post2w | 0.03 | 0.00 | 0.14 | -0.01 | 0.05 |
|  | pre2w - post4w | 0.10 | 0.04 | 0.15 | 0.08 | 0.16 |
|  |  |  |  |  |  |  |
| **Fungi SSI** | pre2w - post2w | -3.31 | -0.43 | -0.54 | 0.83 | 2.81 |
|  | pre2w - post4w | 0.68 | 0.03 | 0.12 | -0.14 | 0.56 |
|  |  |  |  |  |  |  |
| **Fungi Chao** | pre2w - post2w | 77.1 | 40.0 | 67.8 | 84.0 | 26.7 |
|  | pre2w - post4w | 133 | 122 | 91.6 | 57.9 | 12.4 |
|  |  |  |  |  |  |  |
| **Fungi Shannon** | pre2w - post2w | 0.48 | 0.26 | 0.86 | 0.91 | 0.21 |
|  | pre2w - post4w | 0.25 | 0.47 | 0.48 | 0.11 | 0.01 |
|  |  |  |  |  |  |  |
| **Gram-positive** | pre2w - post2w | -214 000 | -108 000 | -74 500 | -58 700 | -255 000 |
|  | pre2w - post4w | -8 770 | 95 900 | 17 100 | 34 200 | -213 000 |
|  |  |  |  |  |  |  |
| **Gram-negative** | pre2w - post2w | 58 900 | -25 300 | -5 210 | 17 000 | 213 000 |
|  | pre2w - post4w | 3 960 000 | 808 000 | 733 000 | 527 000 | 807 000 |
|  |  |  |  |  |  |  |
| **Total fungi** | pre2w - post2w | -18 900 | -2 430 | -8 430 | -2 880 | -7 440 |
|  | pre2w - post4w | 35 800 | 21 100 | 24 000 | 23 700 | 5 270 |
